# Alginate-Based Hydrogels: Recent Progress in Preparation, Property Tuning, and Multifunctional Applications

**DOI:** 10.3390/gels12020182

**Published:** 2026-02-21

**Authors:** Xiaoxu Liang, Shiji Chen, Yuxiong Liang, Miaomiao Wang, Qiao Wang, Dexin Chen, Xiao Ma, Hongyao Ding, Hai-Jing Zhong

**Affiliations:** 1 School of Arts and Sciences, Guangzhou Maritime University, Guangzhou 510725, China; liangxxu@126.com; 2 State Key Laboratory of Bioactive Molecules and Druggability Assessment, Jinan University, Guangzhou 510632, China; chen94599@163.com; 3 School of Big Data and Artificial Intelligence, Guangdong University of Finance & Economics, Guangzhou 510320, China; liangyuxiong@student.gdufe.edu.cn; 4 School of Materials Science and Engineering, Yancheng Institute of Technology, Yancheng 224051, China; wangmiaomiao199113@163.com; 5 School of Civil and Transportation Engineering, Hebei University of Technology, Tianjin 300401, China; qiaowang@hebut.edu.cn; 6 Guangdong Provincial Engineering & Technology Research Center for Corrosion Resistant Materials, Wear and Corrosion Resistant Technology, Institute of Advanced Wear & Corrosion Resistant and Functional Materials, Jinan University, Guangzhou 510632, China; dxchen@jnu.edu.cn; 7 College of Materials Science and Engineering, Nanjing Tech University, Nanjing 210009, China

**Keywords:** alginate, hydrogels, fabrication, biomedical applications, multifunctional materials, wastewater remediation, food industry

## Abstract

Alginate-based hydrogels, derived from brown seaweed, represent biocompatible and biodegradable materials whose properties are systematically controlled through molecular structure (M/G composition), crosslinking strategy, and compositional modification. This review synthesizes recent advances in alginate hydrogel design, encompassing fundamental structural properties, three primary crosslinking approaches—ionic coordination with divalent cations (Ca^2+^, Ba^2+^, Sr^2+^), covalent chemical linkages, and hybrid multi-crosslinking systems—and strategic modification strategies including chemical derivatization, polymer blending, and nanoparticle incorporation. These modifications address inherent limitations of native alginate, namely insufficient mechanical strength and biological inertness, thereby expanding applicability. The review examines applications across biomedical domains (drug delivery, tissue engineering, wound healing), environmental remediation, food industry systems, and emerging technologies including flexible electronics and soft robotics. Advanced fabrication techniques—3D/4D printing, microfluidics, and electrospinning—enable improved architectural control. Current evidence from preclinical and clinical studies demonstrates feasibility in specific applications, while important challenges persist, including predictable degradation kinetics, mechanical property optimization, standardization of characterization protocols, regulatory compliance, and manufacturing scalability. This review aims to provide a systematic assessment of alginate-based hydrogel development and identify areas requiring further investigation to advance clinical translation.

## 1. Introduction

Hydrogels—three-dimensional crosslinked networks retaining substantial water—have emerged as versatile materials for biomedical and environmental applications due to their tunable physicochemical properties and environmental responsiveness [1,2,3,4]. Natural polysaccharide-based hydrogels offer biocompatibility, biodegradability, and ease of modification [5,6,7,8,9,10,11,12]. Alginate, a commercially relevant biopolymer, has applications spanning food, pharmaceuticals, tissue engineering, drug delivery, wound care, and environmental remediation [5,6,13]. Alginate is a linear anionic polysaccharide composed of β-D-mannuronic acid (M) and α-L-guluronic acid (G) residues. The M/G ratio (proportion of mannuronic to guluronic acid units) varies with algal source and extraction method, typically ranging from 0.3 to 3.0. G-rich segments (high G content) form strong, brittle gels through ‘egg-box’ coordination with divalent cations; M-rich regions contribute flexibility and elasticity. The specific M/G ratio fundamentally determines gel mechanical properties, degradation kinetics, and biocompatibility, making it a critical design parameter for tailoring hydrogel performance to specific applications [2,14,15,16,17].

Alginate derives from brown seaweeds (Laminaria, Ascophyllum, Macrocystis) and bacteria (Pseudomonas, Azotobacter), primarily [18,19]. The extraction process, molecular weight distribution, and M/G ratio vary significantly depending on the algal species, harvest season, geographical origin, and processing conditions, substantially affecting the functional properties of the resulting materials [20]. As a material recognized as Generally Regarded as Safe (GRAS) by regulatory authorities including the U.S. Food and Drug Administration (FDA), alginate has found extensive commercial applications across multiple sectors [5,21].

Ionic crosslinking with divalent cations (particularly Ca^2+^) via the ‘egg-box’ mechanism enables rapid gel formation under physiological conditions, offering economic viability and abundant resources for industrial and specialized applications [7,14,22,23]. This unique gelation behavior has facilitated the development of alginate-based materials for applications requiring mild processing conditions, such as cell encapsulation, protein delivery, and injectable therapeutics [24].

Despite these advantages, native alginate hydrogels face critical limitations: insufficient mechanical strength, uncontrolled degradation kinetics, biological inertness (limited cell binding), poor stability, and susceptibility to chelating agents [25,26]. The absence of cell-recognition sequences such as the RGD (Arg-Gly-Asp) peptide motifs in the alginate molecular structure results in poor cell–material interactions, limiting its utility in tissue engineering and regenerative medicine applications [27,28]. Additionally, the mechanical properties of ionically crosslinked alginate hydrogels often fail to match the requirements of specific applications, particularly for load-bearing tissue scaffolds or materials subjected to dynamic mechanical stresses [7,15].

To overcome these critical limitations, researchers have developed comprehensive modification strategies that substantially enhance alginate performance across diverse applications. Physical modification approaches—including strategic polymer blending with biocompatible natural polymers (chitosan, hyaluronic acid, gelatin) or synthetic alternatives (PVA, PEG, PAM)—enable tailored mechanical properties and functional integration without introducing toxic byproducts. Complementary nanomaterial incorporation strategies employing hydroxyapatite (HAP), graphene oxide (GO), carbon nanotubes (CNTs), silica, and metal compounds establish reinforced networks that dramatically enhance mechanical robustness while promoting cellular interactions [7,8,22]. Beyond these physical approaches, systematic chemical modifications—including oxidation, sulfation, phosphorylation, amidation, esterification, graft copolymerization, and click chemistry methodologies—enable precise introduction of functional groups, controlled modulation of degradation kinetics, enhanced bioactivity, and incorporation of stimuli-responsive characteristics that render alginate systems responsive to physiological triggers [6,22]. These integrated strategies transform native alginate from a passive biomaterial into a sophisticated platform capable of addressing complex biomedical and technological requirements.

Advanced alginate hydrogels with stimuli-responsive properties (pH, temperature, ionic strength) enable minimally invasive delivery of therapeutics. Conductive variants show promise in neural/cardiac tissue engineering, biosensing, and flexible electronics [7,24,29,30,31,32]. Advanced alginate-based composite hydrogels with enhanced mechanical properties and biological functionality have shown promise in bone, cartilage, vascular, and neural tissue regeneration [7]. In environmental applications, alginate-based adsorbents have demonstrated excellent performance for heavy metal removal, dye adsorption, and pollutant remediation [27,33]. Furthermore, the integration of alginate hydrogels with emerging technologies such as 3D/4D printing, microfluidics, and artificial intelligence-assisted design has opened new avenues for creating sophisticated functional materials with unprecedented capabilities [6,26].

**Figure 1:**
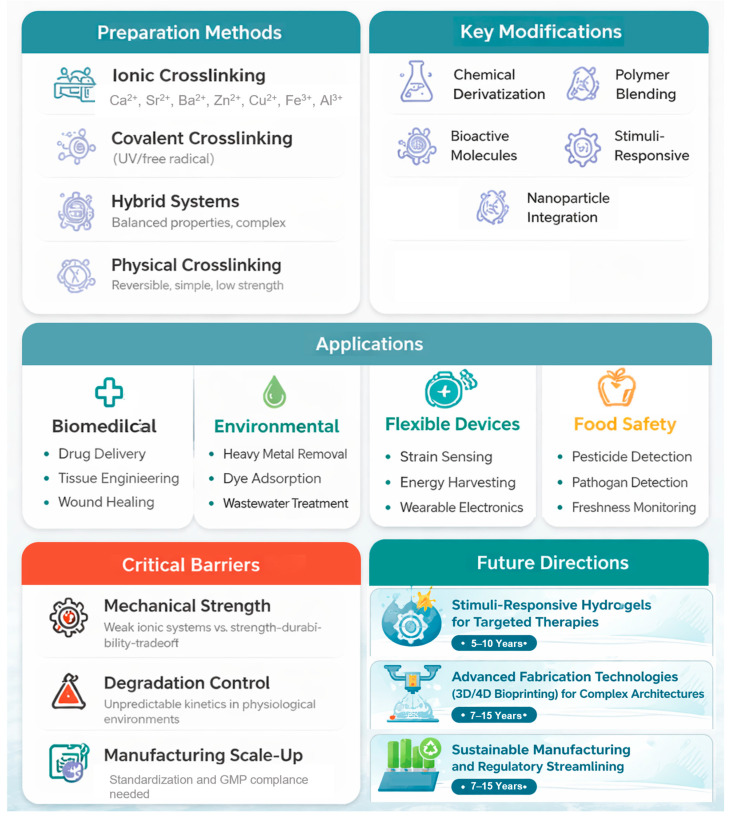
Alginate-based hydrogels: a comprehensive overview of preparation methods, key modifications, diverse applications, critical barriers, and future directions.

This review synthesizes current knowledge on alginate-based hydrogels across six major domains: (1) fundamental properties and preparation, including molecular structure-property relationships, source variations, and diverse gelation mechanisms; (2) advanced crosslinking strategies, encompassing ionic, covalent, hybrid, and physical approaches, with particular emphasis on nanoparticle integration and multi-responsive design; (3) strategic modification approaches including chemical derivatization, polymer blending, and stimuli-responsive element incorporation to overcome native alginate limitations (insufficient mechanical strength, biological inertness, uncontrolled degradation); (4) multifunctional biomedical applications spanning drug delivery, tissue engineering, wound healing, 3D/4D bioprinting, and advanced cell culture platforms; (5) environmental remediation and food safety systems, demonstrating efficacy in heavy metal removal, organic pollutant degradation, pharmaceutical wastewater treatment, pesticide detection, and active food preservation; and (6) emerging technological applications in flexible devices, strain sensing, energy harvesting, soft robotics, electromagnetic shielding, and soil conditioning, as illustrated in Figure 1. Additionally, this review addresses critical challenges impeding clinical translation—mechanical property optimization, degradation kinetics control, manufacturing scalability, and regulatory standardization—while identifying promising future directions including stimuli-responsive hydrogels for targeted therapies, advanced 3D/4D bioprinting architectures, and sustainable manufacturing integrated with regulatory streamlining. By synthesizing state-of-the-art developments and critically analyzing implementation barriers, this review provides researchers, engineers, and practitioners with comprehensive insights into alginate-based hydrogels’ transformative potential for advancing biomedical innovation, environmental sustainability, and technological functionality while establishing a roadmap toward clinical translation and industrial implementation.

## 2. Fundamental Properties and Preparation of Alginate-Based Hydrogels

### 2.1. Chemical Structure and Molecular Composition

#### Basic Molecular Architecture

**Figure 2:**
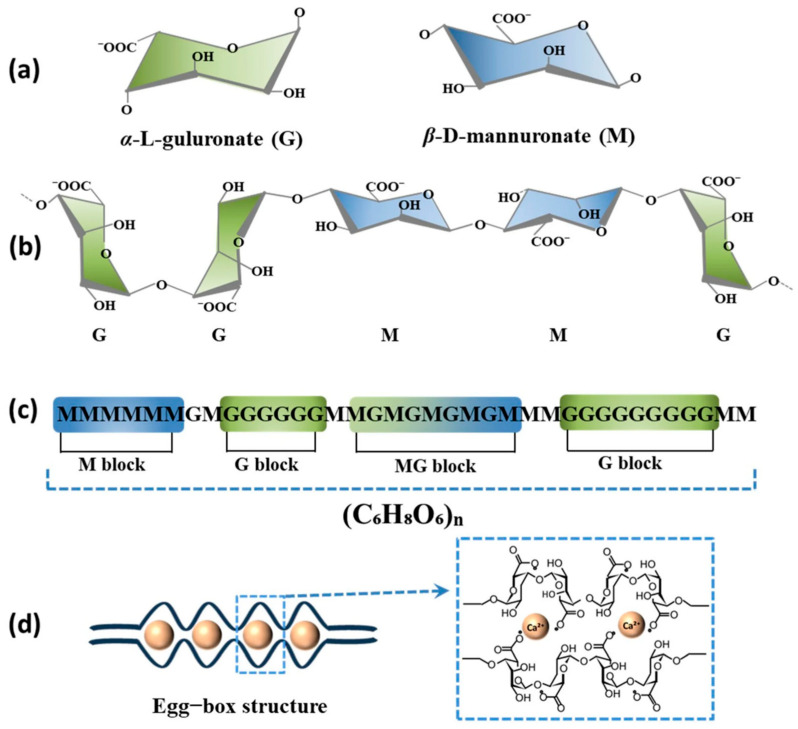
Structural characteristics of alginates. (**a**) Monomer units; (**b**) chain conformation; (**c**) block distribution; (**d**) egg-box structure [16].

Alginate, a linear anionic polysaccharide from brown seaweed and bacteria, comprises β-D-mannuronic (M) and α-L-guluronic (G) acid residues in MM, GG, and MG block arrangements (G content: 30–70% typically; MW: 32,000–400,000 g/mol), as shown in Figure 2 [5,16,34,35,36]. The monomeric carboxyl groups undergo deprotonation at neutral pH, conferring polyanionic character, optimal viscosity at pH 3.0–3.5, and non-Newtonian shear-thinning behavior at reduced concentrations [5,37]. Notably, the M/G molar ratio critically determines mechanical properties: G-enrichment enhances gelling capability and structural rigidity, while M-enrichment improves solution viscosity and flexibility, enabling precise control over final hydrogel characteristics [38].

Alginate forms stable hydrogels via divalent cation interactions (e.g., Ca^2+^, Ba^2+^), generating ‘egg-box’ complexes with G-blocks (as shown in Figure 2d [16]). Acidic conditions induce aqueous swelling [14,34,39,40,41,42,43]. The polymer demonstrates pH-dependent mucoadhesion and exhibits exceptional biocompatibility, biodegradability, antioxidant and anti-inflammatory properties, positioning it as an ideal material for tissue engineering, drug delivery, and wound dressing. While alginate demonstrates antioxidant and anti-inflammatory potential in vitro—including capacity to reduce nitric oxide, reactive oxygen species (ROS), prostaglandin E2, and cyclooxygenase 2—the translation of these properties into in vivo therapeutic efficacy remains incompletely characterized and requires further clinical validation [34,36,44,45,46,47,48,49,50,51,52].

**Figure 3:**
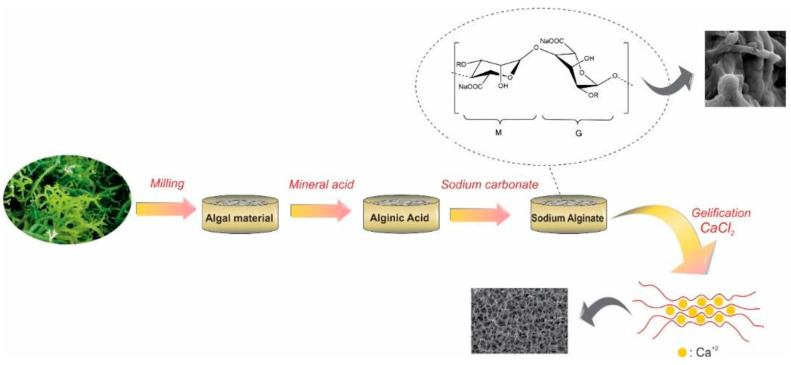
A typical process for the extraction of sodium alginate from brown algae followed by gelation in the presence of CaCl_2_ [34].

Commercial alginate production utilizes seaweed extraction with sodium or calcium chloride solutions (as shown in Figure 3) [34]. Natural sources frequently contain impurities including endotoxins, heavy metals, and proteinaceous contaminants, necessitating rigorous purification protocols to ensure biocompatibility and reproducibility [53]. The selection and implementation of appropriate purification methodologies significantly impact the final product’s quality and suitability for biomedical applications.

### 2.2. Gelation Mechanisms and Crosslinking Strategies

Alginate-based hydrogel networks exhibit three-dimensional architectures that closely mimic natural bio-macromolecular structures, conferring exceptional biocompatibility and enabling non-invasive therapeutic delivery [34]. The physicochemical properties of these hydrogels fundamentally depend on multiple interdependent factors: crosslinking type (ionic, chemical, physical, or hybrid), crosslinker density, polymer composition, and molecular weight distribution. The strategic selection and optimization of these parameters enable precise control over hydrogel performance characteristics. This section comprehensively examines diverse crosslinking methodologies, mechanistic principles, and their consequential effects on hydrogel functionality.

#### 2.2.1. Ionic Crosslinking

Ionic crosslinking with divalent cations—predominantly Ca^2+^—represents the most widely employed alginate-based hydrogel preparation method, establishing characteristic “egg-box” junction sites where divalent cations coordinate with adjacent guluronate block residues [14,15,23,43,54,55,56,57]. This coordination enables instantaneous gelation at ambient temperature and physiological pH, offering substantial advantages over time-intensive covalent approaches. While Ca^2+^ is favored for biocompatibility, alternative cations (Ba^2+^, Zn^2+^, Cu^2+^, Fe^3+^, Al^3+^) induce gelation with distinct mechanical and functional properties; Ba^2+^ typically produces more robust gels due to superior binding affinity for G-block residues, as summarized in Table 1 [15,31,34,56,58,59,60,61,62,63]. The causal chain connecting these parameters operates as follows: (1) M/G composition determines which divalent cations can effectively coordinate with guluronate blocks and the resulting network density; (2) ion species selection produces distinct coordination geometries and binding affinities establishing specific network connectivity patterns; (3) resulting network features—characterized by crosslink density, junction zone distribution, and pore architecture—directly determine macroscopic material behaviors including swelling capacity, degradation kinetics, and mechanical responsiveness; (4) material behaviors (swelling percentage, degradation rate, stress relaxation) measurably propagate into quantified performance outcomes (tensile strength, compressive modulus, cell viability, long-term stability). This integrated mechanistic framework enables systematic understanding of how molecular-level design parameters propagate through network architecture to determine clinical applicability across specific application contexts [15,31,34,56,58,59,60,61,62,63].

Alginate-based hydrogel’s mechanical properties critically depend on gelation rate, temperature, and polymer composition. Higher gelation rates combined with lower temperatures promote homogeneous network structures with enhanced mechanical properties, whereas G-block-enriched alginates yield substantially stiffer hydrogels compared to M-block-enriched variants [23,64]. This composition–property relationship enables rational design of hydrogels tailored to specific mechanical requirements.

In addition, rapid gelation kinetics frequently necessitate controlled methodologies, such as phosphate-buffered systems, to moderate gel formation rates and achieve homogeneous network structures [65,66]. Wang et al. demonstrated that ferrous sulfate (Fe^2+^) doping of sodium alginate hydrogels enhances antimicrobial efficacy and accelerates infected wound regeneration. Fe-doped alginate (Fe-ALG) hydrogels were fabricated via ionotropic gelation with calcium chloride crosslinking, where ferrous sulfate incorporation modulated network properties and imparted antimicrobial functionality. Fe-ALG hydrogels exhibited significantly accelerated wound closure compared to buffer control, unmodified sodium alginate (Na-ALG), and vancomycin-treated groups at both 7- and 14-day timepoints [67].

Recent studies demonstrate the remarkable versatility of metal-incorporated ionic systems [67,68,69]. For instance, Urzedo et al. presents the first report of alginate hydrogels simultaneously incorporating S-nitroso-mercaptosuccinic acid (S-nitroso-MSA, a nitric oxide donor) and green tea-synthesized silver nanoparticles (AgNPs) for synergistic antimicrobial action. Hydrogels are fabricated via ionotropic gelation (CaCl_2_ crosslinking) with glycerol plasticization to enhance mechanical flexibility, while AgNPs averaged 35.3 ± 5.9 nm with zeta potential of −35.5 ± 1.2 mV, confirming stable colloid formation [68]. Additionally, copper alginate (SA-Cu) hydrogels with lower M/G ratios exhibit improved crosslinking density, mechanical strength, and immunomodulatory properties [61]. These metal-enhanced systems exemplify how ionic crosslinking platforms accommodate functional diversification without compromising core gelation mechanisms.

Ionically crosslinked alginate hydrogels exhibit exceptional swelling capacities (1500–4200%), favorable porosity (56–85.20%), high water content (74–94%), variable Young’s modulus (0.99–9.75 MPa), and excellent drug encapsulation efficiency (87–95%) [69,70,71,72,73]. These characteristics render ionic systems particularly suitable for drug delivery and cell encapsulation applications. However, purely ionic crosslinked hydrogels exhibit significant limitations: inadequate mechanical strength (rarely exceeding 1–10 MPa), rapid physiological degradation through ion exchange mechanisms, and insufficient long-term structural stability [69,74,75].

To overcome these mechanical constraints, Ji et al. developed an innovative “reconstruction approach” involving anisotropic drying of pre-gel substrates followed by sequential additional crosslinking and rehydration. This multi-step strategy produced hydrogels with substantially enhanced tensile strengths (8–57 MPa) and elastic moduli (94–1290 MPa), with Fe^3+^ yielding the most robust networks [75]. This approach demonstrates how post-gelation treatment can substantially amplify mechanical performance while preserving the advantages of ionic crosslinking. Additionally, Tordi et al.’s innovation leverages ionically tunable networks through dual-cation crosslinking, wherein Cu^2+^ or Mn^+^ ions establish alginate network structure while systematic Li^+^ doping modulates ionic transport and mechanical properties. Gelatin and alginate solutions were sequentially crosslinked with MCl_2_ (M = Mn or Cu) and LiCl, followed by glycerol–water equilibration. SAXS analysis reveals fundamentally distinct network architectures governing electrochemical performance. Cu^2+^ forms compact, structurally resilient networks (ξ = 3.5–3.9 nm) resistant to Li^+^ perturbation through strong binding affinity. Conversely, Mn^2+^ networks progressively loosen (ξ = 6.3–14.7 nm) as Li^+^ concentration increases via competitive ion exchange, enabling enhanced ionic mobility despite reduced mechanical integrity. This synergistic dual-crosslinking strategy reveals decoupled optimization of mechanical robustness and ionic conductivity—a balance unattainable in single-ion systems: (1). Cu^2+^ pathway: Maintains high mechanical integrity (~1.36 MPa) while Li^+^ doping progressively enhances ionic conductivity through interstitial conduction; (2) Mn^2+^ pathway: Li^+^ reduces gel modulus by >60% (0.15 MPa at Mn025AlgGel) but dramatically improves ionic mobility through network loosening. The demonstration that mechanical robustness and ionic conductivity are independently optimizable via Cu^2+^/Mn^2+^ selection and L^2+^ doping represents a significant mechanistic advancement, enabling rational design of electrolytes with tailored performance profiles [76].

**Table 1:** Summary of recent representative ionic crosslinking of alginate hydrogels [15,31,34,56,58,59,60,61,62,77].

Ionic	G-Block Binding Affinity	Crosslink Density	G′ Modulus (Pa)	Yield Stress	Mechanical Robustness	Recovery (%)	Functional Properties
Ca^2+^	Moderate	Moderate	~0.02–0.03	Moderate	Good gel-like behavior	>86%	Shear-thinning, pseudoplastic behavior; G″ > G″ minimal
Mg^2+^	Moderate–High	Enhanced (ADA-GEL-1.0 Mg)	--	--	33.9 ± 3.7 kPa (Compressive modulus)	--	Cartilage tissue engineering (Optimal)
Sr^2+^	High	High	~0.04–0.05	Low	Excellent gel structure	>86%	Similar to Ca^2+^ but more robust; lower tan(δ)
Ba^2+^	Very High	Very High	~0.08–0.10	Lowest	Superior rigidity; dominant elastic behavior	>86%	Highest viscosity, lowest damping factor, most uniform filaments
Cu^2+^	Moderate–High	0.070–0.18 mol/cm^3^	0.4–2.5 MPa	1200–3500	Enhanced mechanical strength;	--	Cu-Alginate systems: dual antimicrobial + mechanical enhancement
Zn^2+^	Moderate	0.060–0.13 mol/cm^3^	0.2–1.5 MPa	1200–3800	Moderate enhancement; lower binding affinity than Ba^2+^	--	Zn-alginate systems: antimicrobial efficacy (~95%); moderate mechanical enhancement
Fe^3+^	Extremely High	Very High (Rapid)	~0.02–0.03	None observed	Poor homogeneity	~35.3%	Rapid, uncontrolled gelation; heterogeneous network; poor recovery
La^3+^	Very High	High (Rapid)	~0.10–0.15	Low	Highest energy storage	~33.4%	Strong elastic response; highest G′ values; faster gelation than divalent ions
Cs^+^	None		G″ >> G′	None	No hydrogel formation	N/A	Liquid behavior;

#### 2.2.2. Molecular Entanglement-Based Hydrogels

Molecular entanglement forms non-covalent alginate hydrogels through freeze–thaw cycling, hydrophobic interactions or hydrogen bonding interactions, inducing polymer chain association without covalent bond formation. This strategy enables reversible gel properties with simplified preparation protocols compared to chemical approaches, facilitating dynamic functionality essential for responsive applications [78,79,80,81].

For instance, hydrophobically linked poly(AM-co-BA)@NaAlg hydrogels synthesized via cetyltrimethylammonium bromide (CTAB)-mediated micelle formation exhibit exceptional 1611% stretchability, toughness of 403.293 kJ/m^3^, and high strain sensitivity (gauge factor = 27.79 at 900% strain) [81]. Similarly, Abbasi et al. incorporated Pluronic F127 block copolymer into PVA/alginate composites, producing hydrogels with 58.75 MPa tensile strength, 565% elongation capacity, sustained antimicrobial release over 35 h, and significant antibacterial efficacy against clinically relevant pathogens, achieving complete wound closure within 21 days [82]. Aitchison et al. investigated 3D-printed alginate/polyvinyl alcohol (PVA) bioinks for cartilage tissue engineering by characterizing how varying polymer concentrations affect scaffold physical properties. Increasing PVA concentration decreased swelling while increasing degradation rates, demonstrating PVA’s role in modulating hydrogel matrix stability. The highest elastic modulus (0.22 MPa) was achieved with 5% PVA and 20% alginate, approaching native cartilage mechanical properties. These results establish that rationally tuned PVA and alginate concentrations enable bioink development with tailored mechanical properties suitable for cartilage tissue engineering applications [83]. These examples illustrate how hydrophobic interactions and polymer blending enhance mechanical performance beyond native alginate capabilities.

In addition, polyvinyl alcohol (PVA)-incorporated composite systems demonstrate particular promise due to PVA’s inherent hydrophilicity, aqueous affinity, and established biocompatibility [84,85,86]. Jin et al. created a sodium fusidate-loaded triple polymer hydrogel with systematically optimized PVA:polyvinylpyrrolidone (PVP):sodium alginate molar ratio (10:6:1), demonstrating superior mechanical properties (324.7 ± 12.4% swelling, 175.97 ± 16.30 g bioadhesion), sustained pharmaceutical release (71.8 ± 1.3% within 24 h), and enhanced dermal permeation compared to commercial products [87]. Liang et al. developed a water-soluble composite hydrogel combining carboxymethylcellulose sodium (CMC-Na), sodium alginate (SA), and chitosan (CS) to simultaneously achieve moisture self-regulation and anti-adhesion properties—critical requirements previously unmet by conventional burn dressings. The hydrogel forms through thermally induced physical polymer blending via hydrogen bonding and electrostatic interactions between anionic CMC-Na and SA and cationic CS, reinforced mechanically by a polypropylene nonwoven scaffold. This non-permanently crosslinked matrix achieves 99.48 ± 1.03% wound closure over 20 days, exhibits superior water vapor permeability (1810.777 ± 131.679 g·m^−2^·d^−1^), and provides effective inflammatory modulation [88]. These formulations demonstrate how polymer composition optimization achieves synergistic property enhancement.

#### 2.2.3. Chemical Crosslinking

Covalent crosslinking establishes three-dimensional networks via covalent interactions utilizing hydroxyl and carboxyl groups inherent to alginate [47,89]. Unlike ionically crosslinked gels that undergo stress-induced dissociation and plastic deformation, covalently crosslinked gels exhibit elastic deformation, superior mechanical stability, and prolonged structural integrity. Chemically crosslinked alginate hydrogels can be synthesized via multiple mechanisms: free radical polymerization, macromolecular self-crosslinking, and click chemistry approaches [24,90,91,92,93,94,95]. However, potential toxicity of conventional crosslinking agents necessitates careful removal of unreacted chemical species to ensure biocompatibility [18,96].

Investigations into different crosslinking agents and crosslinking density regulation demonstrate that mechanical properties and swelling behavior can be finely tuned [97]. Eiselt et al. demonstrated that covalent cross-linking of sodium alginate with poly(ethylene glycol)-diamines (PEG-diamines) of varying molecular weights overcomes the mechanical limitations of ionically cross-linked alginate hydrogels. Covalent coupling via carbodiimide chemistry (EDC/NHS activation) yields stable, tunable mechanical properties with precise control over cross-linking density. Hydrogel mechanics are tunable through two parameters, (1) PEG molecular weight and (2) cross-linking density, enabling rational design of hydrogels with elastic moduli spanning 9–110 kPa. High cross-linking efficiency (~90%) with minimal unreacted PEG-diamines (0.2–5.3%) validates the synthetic approach. However, unexpected mechanical decline at high PEG loading (>27 wt%) reveals limitations of current rubber-elasticity theory in predicting two-component macromolecular network behavior [98].

Kulkarni et al. developed interpenetrating network (IPN) hydrogel membranes combining sodium alginate (SA) and poly(vinyl alcohol) (PVA), cross-linked with glutaraldehyde (GA), for sustained transdermal delivery of prazosin hydrochloride, an anti-hypertensive agent. The IPN architecture overcomes single-polymer limitations by creating a three-dimensional network where GA acts as a bi-functional cross-linking agent, forming acetal bridges between hydroxyl groups of SA and PVA and aldehyde groups of GA. IPN membranes achieved sustained transdermal drug release over 24 h with significantly extended release kinetics compared to rapid release from pure SA (99.3% at 12 h) or PVA (82.8% at 12 h) alone. Tensile strength increased two-fold in IPNs (3.98–4.53 kg/cm^2^) relative to individual polymers (SA: 2.34 kg/cm^2^; PVA: 2.98 kg/cm^2^), confirming superior mechanical properties through extensive inter-polymer chain linkages [97].

Beyond permanent covalent bonding, Schiff-base chemistry creates pseudo-covalent imine bonds with reversible equilibrium, enabling hydrogel fabrication with dynamic properties and self-healing capabilities [99,100,101,102]. Sodium periodate oxidation of alginate produces alginate dialdehyde (ADA) with reactive aldehyde groups forming Schiff-base linkages with gelatin, chitosan, or proteins, creating hybrid hydrogels with enhanced cell adhesion, modifiable degradation kinetics, and inherent self-healing properties through continuous imine bond uncoupling/recoupling cycles [91,92,93,94,103]. This reversible crosslinking strategy permits dynamic network reorganization, accommodating mechanical stress and promoting tissue integration.

Basu et al. developed a DNA-based nanocomposite hydrogel cross-linked via reversible imine linkages between amine groups on DNA nucleobases and aldehyde groups of oxidized alginate (OA), incorporating silicate nanodisks (nSi) to enhance mechanical properties and self-healing capacity. Oxidation of alginate dramatically enhanced gel strength: storage moduli (G′) increased from 12.59 Pa (21.64% oxidation) to 199.5 Pa (53.76% oxidation) at 2.8% OA concentration, while viscosity rose from 78.1 Pa·s (DNA alone) to 416 Pa·s (DNA + OA). Yield stress increased 15.8-fold and yield strain increased 6.3-fold with oxidation enhancement, demonstrating oxidation degree as the primary mechanical control parameter. The hydrogels sustained simvastatin release over extended periods through tunable imine cross-link dynamics [99]. Additionally, Huan et al. fabricated injectable self-healing hydrogels using oxidized sodium alginate (OSA) and carboxymethyl chitosan (CMCS) incorporating vascular endothelial growth factor (VEGF) and human umbilical vein endothelial cells (HUVECs); in vivo studies in streptozotocin-induced diabetic mice demonstrated sustained glycemic control extending to 100 days [100]. These applications exemplify how Schiff-base chemistry enables sophisticated therapeutic delivery combined with dynamic mechanical functionality [99,100,104].

Alginate undergoes modification via grafting copolymerization with hydrophilic vinyl monomers through radical abstraction mechanisms [105,106,107]. Wang et al. developed semi-interpenetrating polymer network (semi-IPN) superabsorbent hydrogels from sodium alginate-g-poly(sodium acrylate) (NaAlg-g-PNaA) and polyvinylpyrrolidone (PVP), demonstrating enhanced water absorption (1004 g/g), increased swelling kinetics, and pH-sensitive reversible switching behavior. This approach facilitates incorporation of responsive functionality through straightforward chemical modification [105].

Free radical polymerization establishes covalent crosslinks between methacrylate groups, substantially enhancing mechanical properties and providing superior structural control [34,108]. Modified alginate with photocrosslinkable chains enables methacrylate functionalization followed by photoinitiator-mediated polymerization under physiological conditions, allowing spatiotemporal cell or bioactive molecule encapsulation [108,109,110].

Coates et al. prepared methacrylated alginate via reaction with methacrylic anhydride, followed by VA-086 photoinitiator polymerization under 365 nm UV light. Mesenchymal stem cells (MSCs) at 3 × 10^6^ cells/mL demonstrated high initial viability (80%) with negligible statistical differences in metabolic activity compared to calcium crosslinked controls over 10 days [111]. However, chemical photo-initiators (VA-086, VA-044, V-50, Irgacure 1870) exhibit cytotoxicity toward encapsulated cells and bioactive molecules, with prolonged UV exposure substantially compromising cell viability [109,111,112]. To address these limitations, Gao et al. developed dual crosslinked methacrylated alginate (Alg-MA) microfibers using sequential ionic crosslinking with Ca^2+^ followed by UV photocrosslinking. These dual crosslinked systems demonstrated substantially improved swelling control (60% reduction) and dimensional stability (25% weight loss after 7 days in simulated body fluid), effectively minimizing photoinitiator exposure while maintaining mechanical performance [109].

Microwave irradiation provides an environmentally sustainable alternative to conventional thermal processing, facilitating rapid reaction kinetics while minimizing solvent utilization and hazardous byproduct generation [113,114]. Zhao et al. synthesized sodium alginate dimethyl diallyl ammonium chloride (SAD) via microwave-accelerated free radical copolymerization [113], while Chen et al. demonstrated that microwave-assisted drying of calcium alginate beads with volcanic tephra enhanced mechanical stability, surface morphology, and phosphate adsorption capacity [114]. These green chemistry approaches align with sustainability imperatives while maintaining synthesis efficiency.

#### 2.2.4. Hybrid and Multi-Crosslinking Systems

Advanced hydrogel systems employ multiple complementary crosslinking mechanisms to achieve synergistic effects and amplified functionality. This hybrid strategy circumvents limitations inherent to individual crosslinking approaches while leveraging complementary advantages [115,116,117]. Cao et al. synthesized double-network hydrogels combining gelatin methacrylate (Gel-GMA) and OSA via sequential Schiff-base reactions and UV photo-crosslinking, achieving compressive strength of 77.82 ± 6.82 kPa with exceptional biocompatibility (>95% cell viability after 48 h) and effective antimicrobial properties [118]. Sequential crosslinking strategies effectively minimize photo-initiator exposure while maintaining superior mechanical performance, demonstrating the principle that rational crosslinking order enhances outcomes.

Recent innovations extend this principle further. Triple-crosslinked systems incorporating phenylboronic acid-grafted polyethyleneimine (PBA-PEI)-modified gelatin and alginate dialdehyde demonstrate how imine bonds, boronate ester linkages, and calcium-mediated ionic crosslinks collectively enhance self-healing capacity, mechanical strength, and multi-stimuli responsiveness through diverse interaction mechanisms [48,119]. This architectural complexity enables sophisticated control over degradation, mechanical behavior, and functional response to physiological signals.

#### 2.2.5. Nanoparticle Integration and Material Reinforcement

Nanomaterials with exceptional porosity and surface area-to-volume ratios establish interconnected nanofibrous networks within alginate matrices, enhancing mechanical properties while promoting cell adhesion and improving bioink characteristics [119,120,121,122,123,124,125,126,127]. For instance, Cordero et al. systematically investigated how one-dimensional (1D) nanoparticle length affects photo-crosslinked methacrylated alginate (AlgMA) hydrogel properties. Aluminum oxide nanowires (NWs: 21 nm) and nanofibers (NFs: 166 nm) were functionalized with amino groups, fluorescently labeled with FITC, and photopolymerized into AlgMA at concentrations of 0.01–1.0 wt%. At 0.5 wt% loading, nanofibers achieved 8-fold complex modulus increase compared to 2-fold for nanowires, with proportionally greater yield strain reduction. Swelling decreased 2.5-fold with NFs versus minimal change with NWs, attributed to increased hydrogen bonding crosslinking. Activation energy increased significantly at >50% conversion, demonstrating nanoparticle-induced thermal stabilization through hydrogen bonding between surface hydroxyls and alginate carboxylate/hydroxyl groups. Nanofiber superior reinforcement stems from restricted polymer chain mobility and enhanced stress transfer via higher aspect ratio, establishing a dual control mechanism: length-dependent network connectivity and hydrogen bonding interactions [66]. Additionally, single-walled carbon nanotubes (SWCNTs) at 1 wt% demonstrate substantial mechanical enhancements (tensile strength +24.3%, secondary modulus +49.7%, elongation +17.9%) with improved cell adhesion; however, concentrations exceeding 1 wt% may increase cytotoxicity, necessitating careful dose optimization [67].

Nanocellulose forms—cellulose nanocrystals (CNCs) and cellulose nanofibrils (CNFs)—improve pore architecture and nutrient transport while enhancing mechanical properties through reinforcement mechanisms [68,69]. CNCs function as effective shear-thinning additives in bioink formulations, enabling improved printability, whereas CNFs enable high-precision 3D scaffold fabrication maintaining morphological fidelity for extended periods (up to 30 days), demonstrating utility in advanced manufacturing approaches [68,69].

Mesoporous silica nanoparticles (MSNs) and hydroxyapatite nanoparticles (HAPs) enhance mechanical properties and promote cell proliferation through bioactive ion release and surface interactions; 1 wt% HAP-enriched hydrogels achieve porosity exceeding 90% with superior bone regeneration induction capacity. These mineral-based nanomaterials bridge synthetic and biological functionality, facilitating superior tissue–material integration [122].

However, nanoparticle co-modification presents significant challenges including strong intermolecular interactions leading to microporous architecture disruption, decreased effective porosity, and increased enzymatic degradation resistance that counteracts intended benefits. Nanoparticle agglomeration remains a persistent limitation requiring strategic resolution through advanced surface modification and dispersion methodologies [70]. Future approaches should emphasize rational surface engineering, molecular functionalization, and advanced dispersion techniques to maximize nanoparticle functionality while maintaining biocompatibility and structural integrity.

### 2.3. Summary: Crosslinking Strategies and Future Directions for Alginate Hydrogel Development

Alginate-based hydrogel engineering depends critically on crosslinking methodology selection. As detailed in Table 2, ionic approaches enable rapid gelation with excellent biocompatibility but modest mechanical properties; covalent methods deliver superior mechanics with biocompatibility concerns related to photo-initiator toxicity; hybrid systems achieve balanced optimization of competing properties. Successful formulations integrate M/G composition, crosslinking density, bioactive modifications, and manufacturing considerations as an inter-dependent system. The following sections detail application-specific implementations demonstrating how rational design within this framework enables therapeutic utility across multi-field domains.

## 3. Properties and Multifunctional Applications of Alginate-Based Hydrogels

### 3.1. Biomedical Applications

Alginate-based hydrogels leverage biocompatibility, biodegradability, and hydrophilicity for biomedical applications in drug delivery, gene therapy, tissue engineering, and diagnostics, which demonstrate exceptional capabilities [128,129,130]. Recent alginate-based hydrogel systems for drug delivery, tissue engineering, wound healing, and diagnostics are reviewed below.

#### 3.1.1. Advanced Drug Delivery System

Alginate-based hydrogels have emerged as versatile platforms for biomedical applications, effectively addressing the limitations of native alginate through innovative chemical modifications and advanced delivery strategies, as summarized in Table 3 and Table 4 [29,131]. Native, unmodified alginate exhibits limitations including modest mechanical strength (typically <10 MPa tensile strength), variable stability across pH ranges, kinetically unpredictable degradation in physiological environments, and rapid biodegradation—constraints that restrict its utility in demanding applications [132,133,134,135,136,137,138].

**Figure 4:**
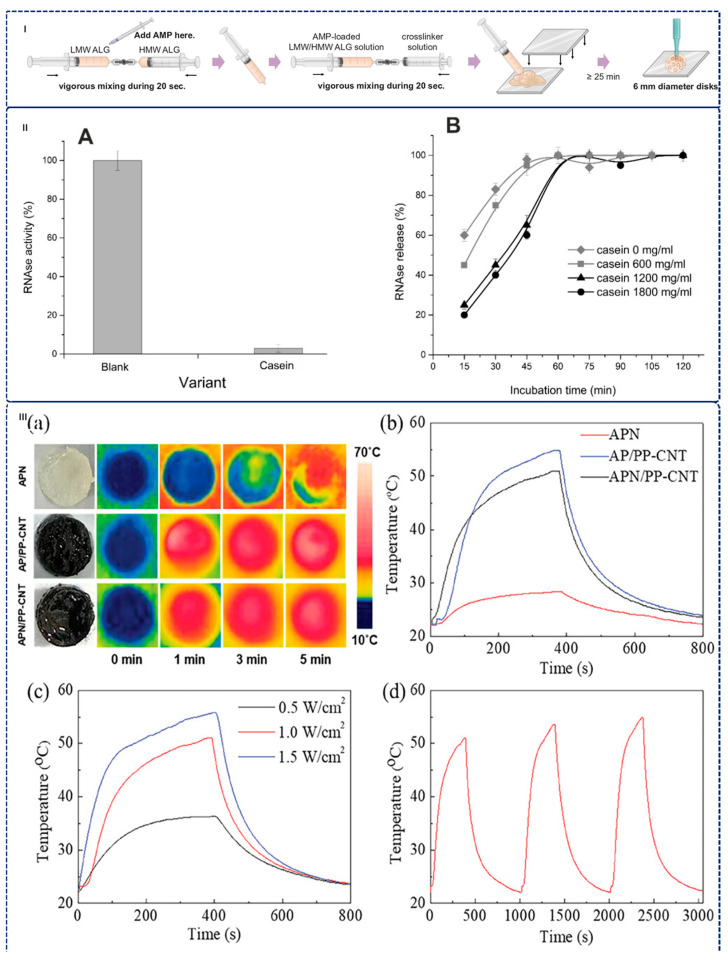
(**I**) Schematic representation of the preparation of alginate-based hydrogels for AMP delivery [140]. (**II**) RNase activity in blank solution (300 µg/mL in 0.01 M PBS pH 7.2) and in solution containing 1 mg/mL beta-casein after ultrafiltration through a membrane filter with a pore diameter of 30 kDa (**A**) and time-course of binase release from alginate hydrogel and alginate hydrogel supplemented by beta-casein (**B**). We took 100% of RNase activity of the loaded protein (calculated as difference between the activity of initial solution in 0.01 M sodium phosphate buffer and resting activity after hydrogel incubation) [143]. (**III**) (**a**) Photothermal effect and (**b**) temperature elevation of hydrogels under NIR-II irradiation (1064 nm, 1.0 W.cm^−2^). Temperature variation in APN/PP-CNT hydrogel (**c**) under NIR-II irradiation with different power densities and (**d**) under cyclic NIR-II irradiation (1064 nm, 1.0 W.cm^−2^) [119].

Recent advances in alginate modifications highlight their potential as sophisticated delivery systems [138,139,140,141,142]. For example, to enhance curcumin’s therapeutic potential—addressing its poor water solubility, rapid metabolism, and minimal oral bioavailability (1%)—Paswan et al. developed sodium alginate/curcumin-PLA (SA/Cur-PLA) hydrogel beads through a novel encapsulation strategy. By combining sodium alginate with polylactic acid and employing calcium chloride as a crosslinker, the formulation achieved multiple advantages: PLA incorporation enhanced gel fraction, crosslinking density, and swelling capacity while providing UV protection for curcumin. Solubility improved dramatically from 0.1 mg/mL to 0.814 mg/mL per 1 mg PLA, and cellular uptake increased with cytocompatibility exceeding 96% at 0.5 mg/mL concentration. The SA/Cur-PLA system demonstrated 81.47% encapsulation efficiency and exhibited intelligent pH-responsive release: complete curcumin release within 12 h under simulated intestinal conditions (pH 7.4), while remaining stable in acidic gastric environments (pH 1.2) for 2 h [139]. Using Design of Experiment (DoE) software to modulate formulation variables including polymer ratios, cross-linker concentration, and oxidation modification (the schematic representation of hydrogel preparation is as shown in Figure 4I), optimized alginate-based hydrogels also could sustain delivery of two antimicrobial peptides (AMPs)—human β-defensin 2 (hBD-2) and PP4-3.1. The optimized hydrogels exhibited high storage modulus (14.79 ± 1.69 kPa), nanometric porous structure (average pore diameter 5.16 ± 6.17 nm), and sustained AMP release over three days with approximately 51.91 ± 5.14% released by 12 h [140]. Similarly, Bogdanova et al. created Ca^2+^-enhanced alginate hydrogel microspheres for prolonged ribonuclease release, maintaining enzymatic activity during sustained delivery (as shown in Figure 4II) [143]. These advancements in drug delivery mechanisms—focusing on diffusion, swelling, and responsiveness—position alginate as a versatile platform for addressing diverse clinical needs [29,131].

**Table 3:** Summary of quantitative performance metrics for recent representative alginate-based hydrogels used in drug delivery.

Hydrogel System	Encapsulation Efficiency (%)	Drug Release (24 h, %)	Cell Viability (%)	Ref.
PNIPAM/PAM/Alg Ca^2+^	87–95 (dexamethasone)	Sustained	>80	[144]
SA/Curcumin/PLA	81.47 (curcumin)	81 (pH 7.4, 12 h)	>96	[139]
Fe-MOF/PTX/Ce6@ALG	Ce6: 97.9%; PTX: 86.17%	Sustained	~93%	[145]
anti-TGF-β@His/Alg	12.3%	~3-fold increase compared to control group	>90%	[146]
Cellulose Nanocrystals + SA	Doripenem: 98.4 mg/g	Sustained	>90%	[138]
GelGMA-OSA/GS	Not specified	Sustained	>95	[118]
CMC-g-poly(AMPS) with SA Nanoparticles (HN system)	Alg-NP: 88.6–93.2%; FA-Alg-NP: 83.8–90.5%	Sustained	Not specified	[147]
OSA/CMCS/VEGF	Controlled	~80% cumulative release within 72 h	>90%	[100]

**Table 4:** Comparison of qualitative features and mechanisms for recent representative alginate-based hydrogels used in drug delivery.

Hydrogel System	Modification Strategy	Key Innovation	Primary Limitation	Bioactivity Enhancement	Ref.
PNIPAM/PAM/Alg Ca^2+^	Dual network polymer	pH- and temperature-responsive	Difficult degradation in short timeframe	Smart responsive delivery	[144]
SA/Curcumin/PLA	Chemical blending with PLA	UV protection, enhanced solubility (0.1–0.814 mg/mL)	Complex multi-component synthesis	Improved bioavailability (1–dramatically enhanced)	[139]
Gelatin-MA/OSA	Sequential crosslinking	Minimal photoinitiator exposure	Requires optimization	Enhanced biocompatibility	[118]
ADA-Gel-Mg	Bioactive ion incorporation	Mg^2+^ tailored release via MgCO_3_	Complex system design	Chondrogenic cell promotion	[148]
OSA/CMCS/VEGF	Self-healing chemistry	Imine bond dynamic properties	Limited long-term in vivo data	Sustained glycemic control	[100]
APN/PP-CNT Nanocomposite Hydrogel	Dual dynamic covalent crosslinking	Multiresponsive stimuli (pH, H_2_O_2_, temperature, NIR-II light)	Limited sustained drug release duration	1. CNT-induced antibacterial activity; 2. PNIPAM thermal contraction triggers burst drug release under NIR-II-induced heating; 3. pH-responsive release 4. H_2_O_2_-responsive	[119]

Li et al. presented a novel multi-responsive nanocomposite hydrogel combining ADA, PEI, poly(N-isopropylacrylamide) (PNIPAM), and phenylboronic acid-modified carbon nanotubes (PP-CNT), with the key innovation being direct incorporation of PNIPAM during hydrogel formation—a previously unreported approach that generates thermal-responsive networks with enhanced performance through dynamic covalent bonds (imine and boronate ester) creating strong polymer–nanomaterial interactions. The hydrogel demonstrates quadruple-stimulus responsiveness (pH-dependent, temperature-dependent with LCST 32 °C, hydrogen peroxide-triggered, and NIR-II light-responsive) with dynamic covalent crosslinks conferring self-healing and shear-thinning capabilities, enabling injectable administration through 18 G needles and homogeneous CNT distribution within the polymer matrix that prevents leakage while yielding a mechanically robust yet flexible delivery platform. As shown in Figure 4III, NIR-II light penetration depth (3 cm) substantially exceeds UV/visible wavelengths, enabling non-invasive deep-tissue applications with superior safety profile (1 W.cm^−2^ for NIR-II vs. 0.33 W.cm^−2^ for 808 nm systems), while PNIPAM contraction at elevated temperatures triggers burst drug release kinetics and neomycin release demonstrates pronounced pH sensitivity with 88.0% release at pH 5.5 (acidic infection environment) versus 26.5% at physiological pH 7.4, enabling targeted delivery to infection sites with minimal systemic exposure for non-invasive on-demand wound infection treatment, transdermal drug delivery, and deep-tissue therapeutic access via minimally invasive injection [119]. However, critical limitations restrict broader applicability: the LCST of 32 °C limits body-temperature applications since physiological temperature (37 °C) exceeds the responsive transition and potentially causes uncontrolled release, necessitating incorporation of acrylic acid or PEG modification to elevate LCST above body temperature; differential pH-sensitivity across hydrogel compositions requires systematic investigation to establish design guidelines for rational formulation selection.

In cancer therapy, alginate hydrogels exemplify the sophistication achieved through multi-mechanism approaches and pH-responsive targeting that exploit distinctions in the tumor microenvironment [149,150]. For example, Zolfagharian et al. developed sodium alginate/xanthan gum nanocomposite hydrogels containing 5-fluorouracil loaded in sulfuric acid-treated kaolin nanotubes, achieving significantly high apoptosis rates in cervical cancer through controlled pH-responsive release—slower at physiological pH 7.4 but markedly accelerated at pH 5.5 [149]. Building on this concept, Parvaresh et al. designed triple-responsive hydrogels from oxidized sodium alginate with Schiff-base bonds and calcium crosslinking, achieving simultaneous redox-pH and glutathione responsiveness for sustained Adriamycin delivery, significantly enhancing anti-cancer activity [150].

Beyond pH-responsiveness, Shanmugapriya et al. engineered EGFR-targeted fucoidan/alginate hydrogels incorporating chlorin e6 for photodynamic therapy in colon cancer, demonstrating improved efficacy through combined targeting and immunoenhancement [151,152,153]. In a more localized approach, Pan et al. demonstrated ICG-alginate hydrogels for photothermal tumor ablation using in situ Ca^2+^/Mg^2+^ stimuli-responsive gelation, while combined ICG-alginate hydrogel plus 808 nm laser treatment achieved remarkable cell killing with only 5.20% and 4.84% viability at power densities of 0.3 and 1 W/cm^2^, respectively. Critically, in vivo fluorescence imaging revealed that ICG in the hydrogel demonstrated substantially slower metabolism and better localization compared to free ICG, which rapidly distributed throughout the body. In vivo PTT resulted in rapid tumor ablation with tumors nearly disappearing within one week, compared to continuous growth in control groups [77]. These multifaceted cancer therapies illustrate the evolution of alginate systems from simple carriers to sophisticated targeting mechanisms that address the complexities of tumor microenvironments [154,155].

#### 3.1.2. Wound Healing: From Passive Dressing to Active Therapeutic Systems

**Figure 5:**
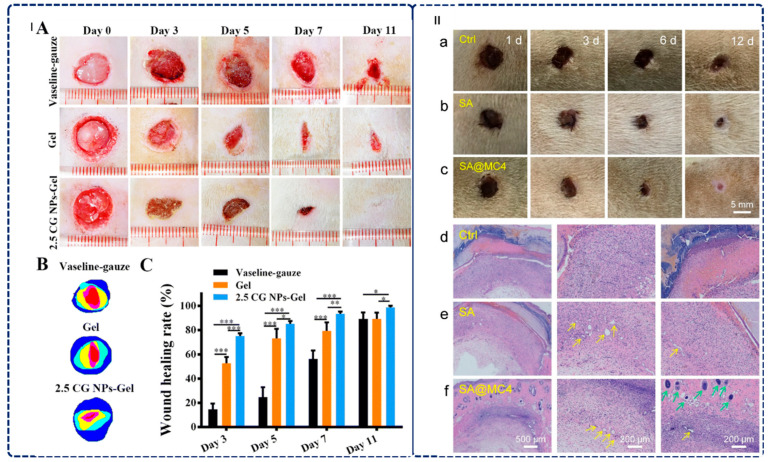
(**I**) In vivo wound healing evaluation of the 2.5 CG NPs-Gel. (**A**) Representative images of wounds in the Vaseline-gauze, Gel, 2.5 CG NPs-Gel groups on days 0, 3, 5, 7 and 11. (**B**) Schematic image of the wound healing process of the different treatment groups. (**C**) Wound healing rate of the Vaseline-gauze, Gel, 2.5 CG NPs-Gel groups on days 3, 5, 7 and 11. * (*p* < 0.05), ** (*p* < 0.01) and *** (*p* < 0.001) [160]. (**II**) Schematic illustration showing formation of Alg-Ph/gelatin hydrogels with capacity of cell attachment based on HRP enzymatic reaction. HRP, horseradish peroxidase [161].

Alginate-based hydrogels have revolutionized wound healing, serving as superior alternatives to traditional dry dressings due to their exceptional capacity to maintain moist environments, absorb exudate, and promote tissue regeneration. These hydrogels can contain up to 99% water and possess a three-dimensional hydrophilic structure, which is essential for effective healing [29,86,156,157,158]. Recent innovations demonstrate their multifunctional potential across various therapeutic mechanisms. For example, Cai et al. created spray double-network hydrogels featuring a dual-layered architecture—an alginate/carboxymethyl cellulose matrix crosslinked by calcium ions for analgesic and hemostatic functions, topped with acidic chitosan for antibacterial properties. This hydrogel polymerizes rapidly within 10 s, allowing for adaptation to irregular wound shapes [159]. In a related study, Cai et al. developed injectable elastic hydrogels crosslinked with curcumin–gelatin nanoparticles, resulting in significantly reduced healing times through synergistic mechanisms, including promoting M2-macrophage polarization, reducing oxidative damage, accelerating collagen deposition, and enhancing angiogenesis (as shown in Figure 5I) [160].

Furthermore, addressing the challenges of infected wounds, Xie et al. developed sophisticated injectable alginate hydrogels that incorporate carboxymethyl chitosan, curcumin–gelatin nanoparticles, and minocycline, ensuring rapid healing of Staphylococcus aureus-infected wounds (as shown in Figure 5II) [161]. These hydrogels utilize sustained-release mechanisms to provide prolonged therapeutic effects while minimizing the need for frequent dressing changes [161]. Extending antimicrobial strategies, Hu et al. developed biocompatible hydrogels that combine alginate with gallic acid-functionalized silver nanoparticles, successfully reducing pro-inflammatory cytokines (IL-6 and TNF-α) while promoting angiogenesis through the upregulation of CD31, α-SMA, and VEGF expression [69]. Tao et al. developed ProΦGel, a pathogen-responsive dual-layer alginate microbead hydrogel that coordinates phages and probiotics for synergistic wound infection therapy, which aims to address the critical challenge of multidrug-resistant Pseudomonas aeruginosa wound infections. Results show exceptional antibacterial performance with phage-only treatment reducing bacterial counts by ~5 logs at 6 h but experiencing regrowth to ~10^8^ CFU/mL by 24 h, whereas phage-probiotic combination achieved complete eradication with no detectable bacteria at 24 h through synergistic pH reduction to ~4.5 that prevents resistant variant proliferation, alongside >80% phage release within 24 h under infection conditions and sustained probiotic viability (10^7^ CFU/mL) for 48 h on ex vivo human skin. These hydrogels could be used as advanced wound dressings for chronic and acute infections caused by antibiotic-resistant pathogens [157]. Moreover, this work establishes a transformative material strategy for coordinating multi-component living therapeutics through spatiotemporal control of sequential release, demonstrating that rational hydrogel engineering can harness the complementary strengths of fast-acting and slow-acting antimicrobial agents to overcome individual limitations and achieve superior infection eradication while promoting tissue regeneration [157].

Alginate biomaterials effectively tackle the challenges of impaired wound healing in chronic wounds through their inherent biocompatibility, biodegradability, and ability to maintain optimal moisture levels—qualities that collectively facilitate tissue regeneration [156]. Emerging technologies, such as piezoelectric and photothermal systems, further address chronic wound complications [162,163]. For instance, Zhang et al. developed injectable PVA-SBQ/oxidized sodium alginate hydrogels incorporating polydopamine particles to achieve enhanced photothermal antibacterial properties, resulting in a 96.45% wound healing rate after 14 days [164].

These innovations illustrate a significant transformation in wound dressings from passive coverings to active therapeutic systems. By incorporating copper ions for antibacterial properties, MXene and zinc ions for combined antimicrobial and conductive functionality, and titanium dioxide nanoparticles for photocatalytic activity, these multifunctional technologies signify a paradigm shift in wound care [165,166]. Alginate-based hydrogels now actively participate in the healing process through controlled drug release, antimicrobial effects, inflammation modulation, and enhancement of tissue regeneration. This integrated approach effectively addresses the multifactorial nature of wound healing, particularly for challenging cases such as diabetic wounds, infected wounds, and chronic ulcers [29]. Their rapid gelation times, injectable delivery methods, sustained therapeutic release, and responsive functionalities position alginate-based hydrogels as next-generation wound care solutions that improve clinical outcomes while reducing the treatment burden [29].

While numerous preclinical studies suggest therapeutic potential, clinical evidence of alginate-based hydrogels remains limited and heterogeneous [156]. For example, a randomized Diabetic foot ulcer (DFU) controlled trial (*n* = 26, 19 completers) comparing sodium alginate-enriched hydrogels with conventional dressings reported reduced inflammatory infiltrates but no significant differences in wound area reduction, PUSH scores, or histological markers of healing [167]. Conversely, specialized formulations incorporating additional bioactive components have shown more promising outcomes, highlighting the importance of composition in clinical efficacy and the need for larger-scale trials [167].

The clinical utility of alginate dressings appears attributable to their biocompatibility and enhanced water absorption capacity (15–17 times higher than conventional dressings) [168]. However, the purported mechanisms involving calcium–sodium ion exchange remain incompletely characterized at molecular and physiological levels, and clinical trials have demonstrated variable efficacy [169]. While some studies suggest reduced dressing change frequency may enhance patient comfort, cost-effectiveness analyses are limited [156]. A specialized calcium alginate formulation incorporating zinc oxide nanoparticles achieved 75% healing rates (vs. 71.1% control) [170] and combining alginate with hyaluronic acid achieved 63.6% healing for venous leg ulcers (vs. 14.3% conventional therapy) [156,171]; however, these represent specialized formulations rather than native alginate, and larger comparative trials are needed to establish definitive cost-effectiveness profiles. Nevertheless, clinical limitations warrant careful consideration; some trials have shown no significant differences between alginate and conventional dressings, and the presence of larger initial wound sizes negatively affects healing outcomes [169]. Moreover, the limited effectiveness of alginate in dry wound environments and a lack of long-term efficacy data necessitate larger-scale trials to assess cost-effectiveness definitively. Despite these limitations, validated clinical applications across various contexts—including diabetic foot ulcers, venous leg ulcers, donor-site injuries, postoperative wounds, pressure ulcers, and PICC insertion sites—establish alginate hydrogels as biocompatible and moisture-retentive solutions that enhance the transition from passive wound support to active healing promotion in real-world healthcare environments. This evolution advances wound care practices toward evidence-based personalized treatment strategies [156].

#### 3.1.3. Tissue Engineering: Biocompatible Scaffolds Mimicking Natural Extracellular Matrix

Alginate-based hydrogels serve as promising biocompatible scaffolds for tissue engineering applications; however, their inherent limitation—the absence of cell adhesion motifs (particularly RGD sequences) [7,14,35]—necessitates strategic chemical modification or composite integration to facilitate effective cell–matrix interactions and cellular differentiation [172,173,174,175]. Despite these constraints, alginate’s exceptional hydrophilicity (74–94% water retention) effectively recapitulates the native tissue microenvironment, enabling optimal nutrient diffusion, signaling molecule transport, and oxygen penetration essential for maintaining cell viability and promoting tissue morphogenesis [29,128,154,176,177,178].

**Figure 6:**
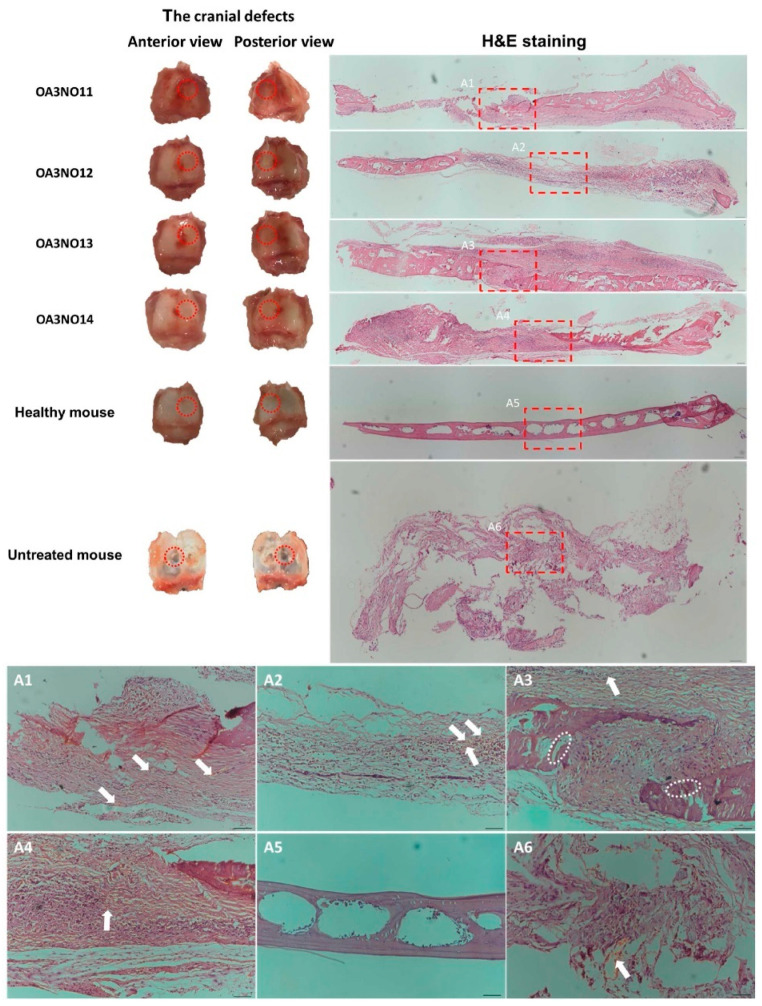
Images showing mouse cranial defects (red dashed circles) and corresponding H&E staining after 4 weeks of follow-up in experimental groups (scale bar 100 μm) and at higher magnification (A1–A6, scale bar 50 μm). White arrow indicates blood vessel and white dashed circle indicates newly synthesized ECM [179].

**Figure 7:**
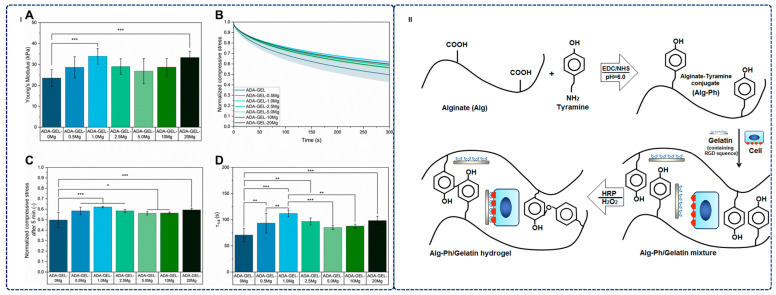
(**I**) Mechanical properties of ADA-GEL-Mg hydrogels showing the effect of MgCO_3_ incorporation on compressive modulus (**A**) and stress relaxation behavior (**B**) with the determined normalized compressive stress after 5 min (**C**) and quantitative stress relaxation time (**D**) of the hydrogels. The data represent mean ± SD of n = 6 specimens for all measurements. To determine the statistical significance, the Holm-Bonferroni test was implemented, with significance levels being * for *p* < 0.05, ** for *p* < 0.01 and *** for *p* < 0.001 [148]. (**II**) Schematic illustration showing formation of Alg-Ph/gelatin hydrogels with capacity of cell attachment based on HRP enzymatic reaction. HRP, horseradish peroxidase [180].

Recent advances demonstrate quantifiable efficacy across diverse tissue types with distinct mechanical and biological outcomes [178,179]. For instance, Vu et al. constructed in situ cross-linked hydrogels using oxidized alginate, N,O-carboxymethyl chitosan, and β-tricalcium phosphate. These hydrogels demonstrated excellent regeneration capacity in mouse skull lesions that exhibited no natural healing ability. Here, β-tricalcium phosphate provided bioactive osteogenic properties (as shown in Figure 6), while oxidized alginate enabled Schiff base crosslinking, enhancing mechanical stability [179]. Zheng et al. developed interpenetrating polymer network hydrogels from alginate and silk protein that incorporated bioactive mesoporous glass as a pore-forming agent. This modification not only enhanced the osteogenic differentiation of bone marrow mesenchymal stem cells but also facilitated controlled bioactive ion release to stimulate bone formation [178]. For further mechanical property improvement, Schöbel et al. developed magnesium-enriched alginate dialdehyde-gelatin hydrogels that incorporated magnesium carbonate at concentrations ranging from 0.5 to 20 mg/mL. These hydrogels achieved dual crosslinking through calcium ions and microbial transglutaminase, enhancing pore sizes to 100–300 μm. Additionally, the effective modulus increased from 23.5 ± 4.0 to 33.9 ± 3.7 kPa (as shown in Figure 7I) while promoting chondrogenic cell proliferation; however, the mechanical properties remained substantially lower than those of native cartilage (0.5–1.5 MPa), and elevated pH from higher concentrations potentially affects cell viability [148].

Injectable hydrogels offer significant clinical advantages for tissue engineering through in situ gelation capabilities, reducing patient morbidity and enabling personalized delivery compared to conventional surgical implantation [154,177,178]. Recent advances exemplify their potential: Shojarazavi et al. developed injectable hydrogels combining alginate with modified decellularized cartilage ECM and silk nanofibers, creating interpenetrating polymer networks that provide both structural support and biochemical cues for tissue regeneration [181]. Similarly, Morshedloo et al. designed injectable alginate hydrogels with phenol segments (Figure 7II) that maintained chondrocyte viability during month-long subcutaneous implantation in rats, with phenol units enhancing mechanical properties and facilitating enzymatic crosslinking [180]. These formulations demonstrate the versatility of alginate-based systems as injectable, biocompatible scaffolds for diverse tissue engineering applications. The chemical and physical modifiability of alginate, combined with integration of bioactive molecules and nanomaterials, offers substantial opportunities for next-generation scaffolds [182]. The minimally invasive nature of injectable systems reduces patient discomfort and recovery time, positioning alginate-based hydrogels as particularly attractive for clinical translation and personalized medicine [173,174,176,182].

Three-dimensional bioprinting of alginate hydrogels represents a transformative fabrication approach for creating patient-specific tissue scaffolds. Singh et al. demonstrated this potential using sodium alginate/58S bioactive glass ink systems, where the ABG10 formulation (10 wt% bioactive glass) achieved superior 3D printing accuracy (>90% versus 30–40% for pure alginate), substantially enhanced mechanical properties (storage modulus increased from 767 to 13,604 Pa; compressive strength improved by 58%), and exhibited excellent cytocompatibility [182]. In vascular engineering, Zhang et al. employed triaxial bioprinting to achieve 3.16 ± 0.10 kPa moduli [183], and Barrs et al. developed peptide-functionalized bioinks that promoted progressive microvascular invasion depths from 200 to 650 μm [184]. Across diverse tissue types—bone, cartilage, skin, neural, and vascular—3D-bioprinted alginate hydrogels have demonstrated cell viabilities of 80–95% with tunable mechanical properties spanning kilopascals to megapascals. Collectively, these advances position alginate-based hydrogels as indispensable materials for regenerative medicine, demonstrating broad applicability across diverse tissue types via advanced 3D bioprinting technologies that connect laboratory innovations with clinical translation [183,184,185,186,187,188,189,190,191,192,193]. However, clinical translation remains limited, with most evidence derived from in vitro and small animal models; long-term in vivo stability and functional integration require further investigation [121,194,195].

Building on these advances, four-dimensional printing extends alginate’s capabilities through programmable spatiotemporal control of structural transformations in response to physiological stimuli. This enables dynamic scaffold design that improves extracellular matrix deposition and facilitates seamless implantation with reduced invasiveness. Díaz-Payno et al. exemplified this approach in cartilage engineering using bilayer hydrogels exhibiting controlled self-bending upon hydration; scaffolds maintained over 90% cell viability and promoted chondrogenic differentiation with sustained extracellular matrix deposition over 28 days [196]. Similarly, Lv et al. developed 4D-printed alginate-gelatin methacrylate scaffolds infused with mineralized collagen for bone regeneration, achieving significantly enhanced bone formation in cranial defect models (bone mineral density: 0.46 g/cc versus 0.32 g/cc in controls) with improved vascularization indicated by elevated CD31 and osteocalcin expression [197]. These developments underscore that 4D-printed alginate-based hydrogels represent a promising strategy for regenerative medicine, combining programmable biomaterial responses with clinically relevant tissue regeneration outcomes [196,197,198,199,200,201,202].

**Figure 8:**
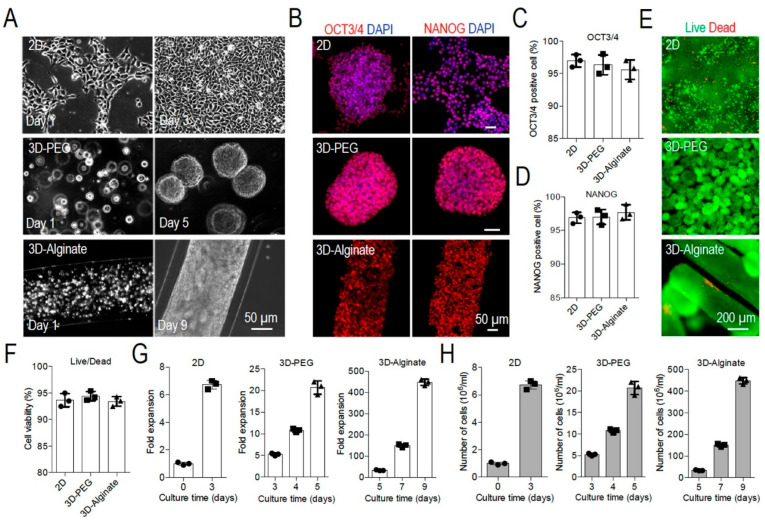
Comparison of hPSC expansion in three culture systems. (**A**) Phase image of hPSC expansion in 2D, 3D-PEG, and 3D-alginate culture systems. Scale bar, 50 μm. (**B**) Immunostaining of the pluripotency markers OCT3/4 and NANOG in 2D, 3D-PEG, and 3D-alginate culture systems. Scale bars, 50 μm. (**C**,**D**) Statistical analysis of OCT3/4- and NANOG-positive cells in 2D, 3D-PEG, and 3D-alginate culture systems. (**E**,**F**) Live-/dead-cell staining and statistical analysis of harvested cells from 2D, 3D-PEG, and 3D-alginate culture systems. Scale bar, 200 μm. (**G**,**H**) When seeded at 1.0 × 10^6^ cells/mL, about 7-fold expansion occurs to yield ∼7 × 10^6^ cells/mL on day 3 in the 2D culture system; about 5-, 10-, and 20-fold expansion, yielding ∼5, 10, and 20 million cells per milliliter of the hydrogel on days 3, 4, and 5, respectively, is achieved in the 3D-PEG culture system; and about ∼30-, 150-, and 480-fold expansion occur to yield ∼30×, 150×, and 480 × 10^6^ cells/mL on days 5, 7, and 9, respectively, in the 3D-alginate culture system. Data are represented as mean ± SD (*n* = 3) [203].

Alginate-based hydrogels have emerged as valuable three-dimensional cell culture platforms due to their biocompatibility, tunable properties, and ability to replicate in vivo tissue microenvironments [193,203,204,205]. Wang et al. conducted a comprehensive comparative analysis demonstrating that three-dimensional alginate hydrogel cultures significantly outperformed conventional two-dimensional monolayers in supporting human pluripotent stem cell-derived endothelial cells across multiple parameters (Figure 8) [203]. Zhang et al. further advanced this application by developing a multi-well plate system for fabricating alginate hydrogel microspheres through gravity-driven gelation combined with bent-capillary-centrifugal dynamics. This approach produced microspheres with exceptional sphericity (Φ = 0.96) and monodispersity (PDI = 0.94%), supporting in situ three-dimensional cell culture with cell viability exceeding 85% [204]. The platform enables high-throughput drug susceptibility testing, single-cell encapsulation, and accelerated drug development validation, demonstrating the versatility of alginate systems for accelerated drug development and personalized medicine applications [204].

Overall, these integrated approaches establish alginate-based hydrogels as versatile biocompatible platforms for regenerative medicine, demonstrating broad multi-tissue applicability through advanced fabrication technologies that effectively bridge laboratory innovations with clinically translatable regenerative solutions.

#### 3.1.4. Regulatory and Safety Requirements for Clinical Translation

Overall, successful clinical translation of alginate-based hydrogels necessitates systematic evaluation of some interconnected safety parameters, each requiring standardized characterization and risk mitigation strategies. (1) Photo-initiator cytotoxicity and UV exposure management represent paramount concerns: conventional photo-initiators (VA-086, VA-044, Irgacure 1870, V-50) exhibit dose-dependent cytotoxicity toward encapsulated cells and bioactive molecules, with prolonged UV exposure (>45 s at high Alg-MA concentrations ≥ 4% *w*/*v*) significantly impairing cell viability and proliferation; residual photo-initiator concentrations must be minimized through sequential crosslinking strategies (e.g., ionotropic pre-crosslinking followed by photo-curing), visible-light activation methodologies, or alternative biocompatible photo-initiators to achieve >85% post-fabrication cell viability [109,111,112]. (2) Metal-ion leaching from functional systems (Cu^2+^, Zn^2+^, Fe^3+^, Ba^2+^) poses secondary risks despite enhanced antimicrobial/mechanical properties: uncontrolled ion release in physiological environments requires rigorous biocompatibility assessment, chelation strategy validation, and quantification of leaching kinetics to prevent systemic accumulation [15,31,34,56,58,59,60,61,62,69,74,75]. (3) Protein adsorption and biofouling in blood-contacting applications may compromise long-term device performance, though alginate’s hydrophilic nature and anionic character provide inherent resistance to nonspecific protein binding compared to synthetic polymers [25,26,27,28]. (4) Innate immune and inflammatory responses necessitate careful characterization: while alginate exhibits documented antioxidant and anti-inflammatory properties (reducing nitric oxide, reactive oxygen species, prostaglandin E_2_, and cyclooxygenase-2), natural source contaminants (endotoxins, heavy metals, proteinaceous material) require rigorous purification protocols to ensure batch-to-batch consistency and minimize immune activation [34,36,44,45,46,47,48,49,51,52]. (5) Post-sterilization performance degradation remains underexplored but critical: gamma irradiation, ethylene oxide (EtO), and plasma sterilization methods may compromise mechanical properties, electrical conductivity in conductive hydrogels, and adsorptive capacity in remediation applications through crosslink disruption and polymer chain scission. Standardized sterilization protocols validated across representative formulations are essential [1,20,166,206]. (6) Cold-storage stability (particularly 4 °C conditions used in clinical manufacturing and distribution) requires documentation: certain alginate systems demonstrate structural integrity exceeding four weeks at refrigeration temperatures, though comprehensive data on moisture loss, microbial colonization kinetics, and oxidative degradation across formulation variants remain limited [2,3,88,207]. These parameters should constitute a standardized regulatory checklist for all preclinical-to-clinical transition studies, with specific acceptance criteria defined through cross-disciplinary consensus involving material scientists, regulatory bodies (FDA, EMA), and clinical investigators to facilitate streamlined regulatory approval pathways and ensure patient safety [5,21].

#### 3.1.5. Summary

Alginate-based hydrogels demonstrate significant potential in biomedical applications, including controlled drug delivery, tissue engineering, wound healing, and diagnostic platforms. Despite challenges in mechanical properties and degradation control, ongoing advances in chemical modifications and fabrication technologies continue to expand their utility. Future directions emphasize stimuli-responsive systems for personalized medicine and regenerative therapies.

### 3.2. Wastewater Treatment

Alginate-based hydrogels offer sustainable environmental remediation through inherent biodegradability, abundant hydrophilic functional groups (–OH and –COOH), and versatile multi-mechanism adsorption capabilities [208,209,210,211,212]. These materials efficiently remove diverse contaminants via electrostatic interactions, ion exchange, surface complexation, and hydrogen bonding, delivering comprehensive single-stage treatment and eliminating the need for sequential operations typically required by conventional approaches. This section synthesizes recent advances in alginate-based hydrogels for wastewater treatment.

#### 3.2.1. Removal of Metal Ions

**Figure 9:**
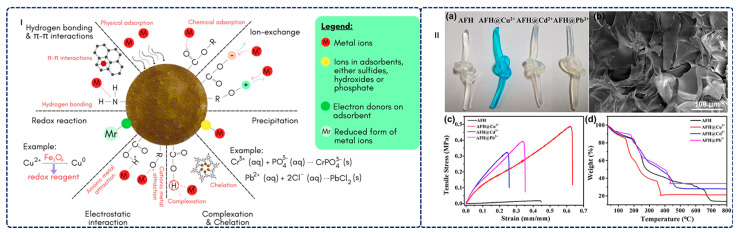
(**I**) Mechanisms of heavy metal ions removal [208]. (**II**) (**a**) Optical images of AFH without and with heavy metal ions adsorbed, (**b**) SEM image of AFH, (**c**) strain–stress curves of AFH with Cu^2+^/Cd^2+^/Pb^2+^ ion adsorbed, (**d**) TGA curve of without and with heavy metal ions adsorbed [215].

Heavy metal ion removal has received considerable attention due to the remarkable efficacy of alginate-based hydrogel composite systems, which employ multiple removal mechanisms as illustrated in Figure 9I [208,209,210,211,213,214,215]. Quantitative performance metrics, mechanistic and design features for recent representative alginate-based hydrogels used in heavy metal removal are summarized in Table 5 and Table 6. For example, MXene/polyaniline/sodium alginate composites achieve impressive adsorption capacities of 255.81 mg/g for Cu(II) and 352.76 mg/g for Hg(II) through integrated mechanisms including physical adsorption, electrostatic attraction, ion exchange, complexation, and redox reactions [214]. Similarly, Kong et al. developed a calcium ion-induced sodium alginate fibroid hydrogel (AFH) that exhibits exceptional performance with saturated adsorption capacities of 315.92 mg/g for Cu^2+^, 232.35 mg/g for Cd^2+^, and 465.22 mg/g for Pb^2+^. The AFH’s three-dimensional network structure enables efficient ion retention, with optimal performance achieved at pH 3.0–4.0 and 303 K [215]. Notably, the hydrogel’s mechanical strength increases after metal ion adsorption, facilitating facile recovery and reuse (as shown in Figure 9II). However, performance may diminish under conditions of variable ionic concentrations and complex multi-ion environments [215].

Beyond these systems, waste valorization approaches demonstrate significant promise. Cross-linked alginate composites incorporating rice husk ash, graphene oxide, and chitosan nanoparticles successfully removed lead ions at 242.5 mg/g, illustrating the potential of utilizing agricultural byproducts for environmental remediation [216]. Temperature-responsive systems further enhance removal efficiency; Wang et al. incorporated poly(N-isopropylacrylamide) into alginate/carboxymethyl cellulose matrices (SCP hydrogel), enabling Pb(II) adsorption at 311 mg/g below the lower critical solution temperature with triggered desorption above this threshold. These materials maintained 73–85% efficiency retention across multiple adsorption–desorption cycles, demonstrating practical viability for dynamic remediation applications [217].

**Table 5:** Summary of quantitative performance metrics for recent representative alginate-based hydrogels used in heavy metal removal.

Hydrogel System	Target Ion(s)	Adsorption Capacity (mg/g)	Removal Efficiency (%)	pH Optimum	Recyclability (Cycles)	Ref.
CL-ARCG-CNP	Pb(II)	242.5	95.2	Not specified	5	[216]
SA/CMC/PNIPAM (SCP)	Pb(II)	311	>91%		8	[217]
PANI@SA/SNM	Cu(II)/Hg(II)	255.81/352.76	Not specified	3–7	8	[214]
SA/MXene/CoFe_2_O_4_	Cu(II)	234.3 (magnetic enhanced)	Enhanced	Not specified	Reusable	[218]
GAHMs	Pb(II)/Cr(III)	327.9/118.6	Not specified	5.0/6.0	5	[219]
Ni-CaAlg Beads	NO_3_^−^/PO_4_^3−^	169.5/238.1	>70 retention	Broad range	10	[220]
AFH	Cu^2+^/Cd^2+^/Pb^2+^	315.92/232.35/465.22	Optimized	3.0–4.0	Reusable	[215]

**Table 6:** Comparison of mechanistic and design features for recent representative alginate-based hydrogels used in heavy metal removal.

Hydrogel System	Primary Adsorption Mechanisms	Design Innovation	Long-Term Stability	Regeneration Method	Ref.
CL-ARCG-CNP	Electrostatic + complexation	Waste valorization (rice husk, GO)	Requires field testing	Chemical regeneration	[216]
PANI@SA/SNM	Physical adsorption, ion exchange, redox	Multi-mechanism composite	Limited environmental testing	Solvent extraction	[214]
AFH	Ion retention via 3D network	Mechanical strength enhancement post-adsorption	Variable in multi-ion environments	Easy recovery and reuse	[215]
Ni-CaAlg Beads	Multiple active sites, electrostatic	Enhanced surface area	Potential Ni leaching	Regeneration optimization needed	[220]
SA/CMC/PNIPAM	Temperature-responsive Pb(II) release	Interpenetrating network structure	73–85% efficiency retention	Thermal desorption	[217]
GAHMs	Electrostatic attraction, Ion-exchange	Porous membrane structure, Synergistic integration of GO with SA	retention rate after 5 cycles: Cr(III): 56.0%; Pb(II): 93.0%	Desorbed with HCl	[219]

#### 3.2.2. Removal of Organic Pollutants

Significant advances have been achieved in organic dye elimination through alginate-based hydrogel systems [221,222]. Hierarchical microgels composed of sodium alginate, polyvinyl alcohol, and zeolitic imidazolate framework-8 (ZIF-8) simultaneously remove both anionic and cationic dyes with adsorption capacities of 180 mg/g for methyl orange and 210 mg/g for methylene blue, demonstrating the versatility of alginate-based materials for diverse contaminant profiles [222]. Beyond conventional calcium coordination crosslinking, Wang et al. demonstrated a paradigm shift from calcium to barium alginate (BA) as a skeleton coating material for graphene oxide (GO) and bentonite-derived composites. Barium’s sp^2^d^2^ hybridization enables 6-fold coordination with alginate G/M units, exposing significantly more adsorption sites than calcium’s planar tetragonal geometry and directly accounting for enhanced performance. This dual-component synergy—bentonite’s cation-exchange capacity combined with GO’s π-conjugated structure—provides complementary electrostatic and hydrogen bonding interactions driving superior dye removal. BA/GO membranes achieved 1011.3 mg/g adsorption capacity, while 3D-BA hydrogels reached 710.3 mg/g, substantially surpassing calcium alginate composites (150.6–181.8 mg/g). Filtration efficiency remained stable at ~75% at high dye concentrations (1000 mg/L), and surface area increased 43-fold with GO incorporation (109.25 m^2^/g vs. 2.55 m^2^/g for pure BA). Capacity retention exceeded 85% after four regeneration cycles, demonstrating excellent reusability. However, future investigations should address long-term Ba^2+^ leaching behavior and performance in real wastewater matrices [223].

Pharmaceutical wastewater remediation presents critical challenges, as antibiotic contamination disrupts beneficial microbial communities and generates toxic byproducts, exacerbating global antibiotic resistance [224,225]. Alginate-based systems effectively address multiple antibiotic classes—tetracyclines, sulfonamides, macrolides, and fluoroquinolones—through diverse adsorption mechanisms [226,227,228,229,230,231,232,233,234]. For instance, sodium alginate/polyvinyl alcohol beads achieve tetracycline removal at 121.6 mg/g via hydrogen bonding and electrostatic interactions [231], while ferrous ion-induced cellulose nanocrystals/alginate composites substantially enhance capacity to 741.66 mg/g through surface complexation and cation bridging [232]. Notably, photocatalytic systems integrating Fe-modified graphitic carbon nitride with alginate achieve 99.18% sulfamethoxazole degradation within 40 min through active pathways while preventing photocatalyst agglomeration [233]. Activated carbon/alginate composites further extend applicability to pharmaceuticals, reaching adsorption capacities of 916.3 mg/g for almotriptan and 588.56 mg/g for paracetamol, underscoring alginate’s adaptability to varied pharmaceutical compounds [234].

#### 3.2.3. Removal of Phosphorus

Phosphorus pollution from fertilizers, manure, and detergents represents a critical environmental concern, driving eutrophication and contributing to significant health complications including bone degradation and renal dysfunction [235,236,237]. Alginate-based materials are particularly effective for phosphate remediation due to their distinctive capillary action, swelling capacity, and abundant functional groups that enable synergistic removal mechanisms [220,238].

Zinc oxide/zinc iron layered hydroxide/alginate composites (ZnO/LDHs/Alg) achieved phosphate removal capacity of 17.06 mg/g with desorption efficiency of 96.81%, while enabling simultaneous composite restoration for sustainable and repeated environmental application [238]. Nickel-doped calcium alginate beads (Ni-CaAlg) substantially enhanced performance to 238.1 mg/g [220]. Advanced systems incorporating zirconium/iron-embedded chitosan/alginate hydrogel beads (Zr/Fe/CS/Alg) and cellulose/lanthanum alginate microspheres (CSPGs-La) demonstrated maximum adsorption capacities of 221.72 mg/g [239] and 159.5 mg/g [240], respectively. Notably, these systems maintained superior selectivity with 73% efficiency retention after six adsorption–desorption cycles, demonstrating both efficacy and sustainability for prolonged wastewater remediation applications [239,240,241].

#### 3.2.4. Performance Enhancement Through Material Integration

Strategic incorporation of advanced materials synergistically enhances alginate-based system performance by circumventing inherent limitations [242,243]. Graphene oxide (GO), a two-dimensional carbon material, exhibits exceptional properties for adsorption applications, including elevated specific surface area, superior electrical conductivity, tunable surface chemistry, and enhanced mechanical strength [219,244]. Similarly, MXene [126,245] and MoS_2_ nanomaterials [242,246] demonstrate comparable advantages. Coupling two-dimensional materials with polymeric matrices improves membrane structural stability through precise layer spacing control, which governs pore architecture and facilitates efficient ion or molecular separation [209].

Jia et al. exemplified this approach by fabricating composite membranes combining sodium alginate with graphene oxide via double crosslinking methodology, achieving enhanced separation performance [244]. In parallel, Song et al. constructed nanofiltration membranes featuring α-Cu(OH)_2_ nanosheets as structural reinforcement and sodium alginate as the polymer matrix (brick-and-mortar architecture), attaining 99.92% dye retention for rose bengal with sustained stability over 48 h while effectively eliminating both Gram-positive and Gram-negative bacteria [247].

MXene nanosheets, characterized by oxygen-containing functional groups and lamellar architecture, substantially strengthen sodium alginate composites while mitigating functional ion leaching [218,248]. MXene/alginate composites achieved Cu(II) adsorption capacity of 87.6 mg/g, with magnetic field-enhanced variants achieving 234.3 mg/g in sodium alginate/MXene/CoFe_2_O_4_ beads developed by Ren et al. [218].

Addressing MoS_2_ hydrophobicity and agglomeration challenges in aqueous environments, Yao et al. incorporated alginate-derived biochar during synthesis, generating biochar-MoS_2_ composites with significantly improved aqueous dispersibility and adsorption performance. This composite exhibited enhanced hydrophilicity and superior adsorption capacities for methylene blue (99.61 mg/g), basic fuchsin (86.83 mg/g), and Cu(II) (60 mg/g), substantially outperforming pristine MoS_2_ [242].

#### 3.2.5. Summary

Alginate-based systems provide comprehensive solutions for remediating heavy metals, organic pollutants, and pharmaceutical contaminants, demonstrating substantial potential for sustainable environmental management. Mechanistic investigations confirm that most systems follow pseudo-second-order kinetics and conform to Langmuir isotherm models, indicating chemisorption-dominated processes [150,209,234,249]. Future research should prioritize optimizing these systems for field-scale applications while enhancing long-term stability and regeneration efficiency to facilitate practical deployment in wastewater remediation.

### 3.3. Flexible Devices

Recent advancements in flexible devices demonstrate the promising potential of alginate-based hydrogels for strain sensing and energy harvesting applications [250]. As natural polyelectrolytes, alginate hydrogels provide an excellent foundation for creating multifunctional materials with sophisticated conductive networks and enhanced mechanical performance. The strategic integration of conductive polymers, carbon nanomaterials, and stimuli-responsive components enables dynamic responsiveness to external stimuli while maintaining superior mechanical stability and electrical conductivity. Consequently, alginate-based hydrogels have attracted significant attention for their contributions to flexible electronics and soft robotics [251]. This section examines the latest developments, capabilities, and challenges associated with alginate-based flexible devices.

#### 3.3.1. Alginate-Based Hydrogel Strain Sensing and Advanced Material Architectures

**Figure 10:**
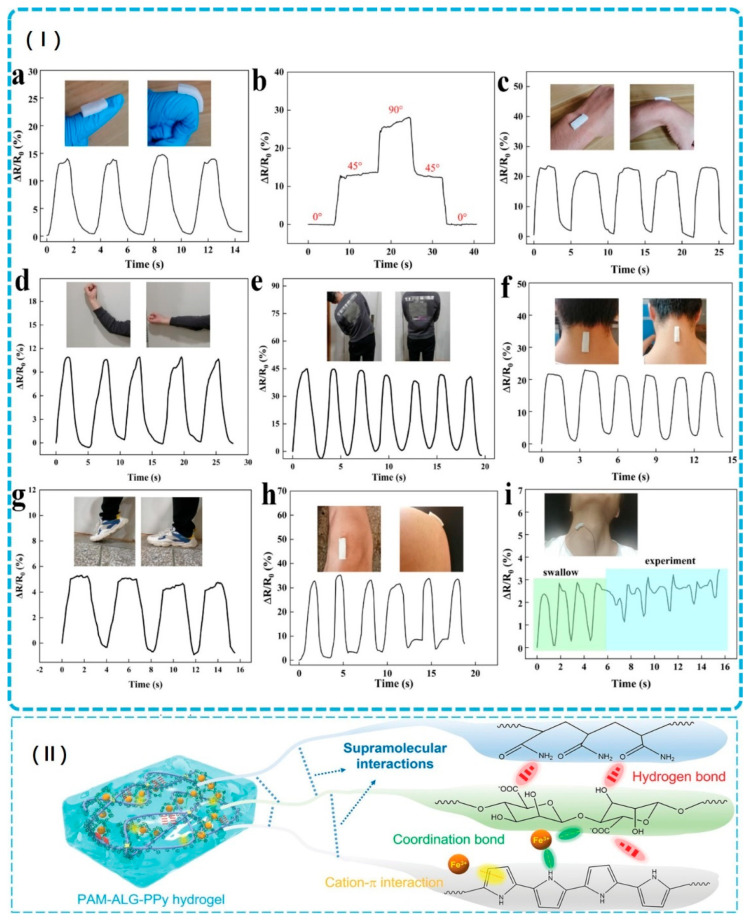
(**I**) Hydrogel for human motion detection. Human movement was detected using SZPM hydrogel sensors: (**a**) fingers, (**b**) fingers bent at different angles, (**c**) wrist, (**d**) elbow, (**e**) waist twisting, (**f**) neck bending, (**g**) ankle, (**h**) knee bending, and (**i**) throat swallowing and speaking [253]. (**II**) Interactions between various polymer networks in PAM-ALG-PPy hydrogel [256].

Alginate-based hydrogels represent outstanding candidates for strain sensor applications due to their sophisticated conductive network design and mechanical reinforcement capabilities [250,252]. Quantitative performance metrics, mechanistic and design features for recent representative alginate-based hydrogels used in strain sensing are summarized in Table 7 and Table 8. Su et al. exemplified this by developing a conductive hydrogel combining chitosan and sodium alginate via an eco-friendly approach. Hydrogen bonding between these polymers, coupled with Ca^2+^ crosslinking, created a dual physical crosslinking network functioning as a sensitive ionic conductor generating electrical signals in response to motion [252]. As a natural polyelectrolyte, alginate provides abundant conductive ions enabling high-sensitivity monitoring of weak physiological signals—essential for biomedical applications (as shown in Figure 10I) [250,253,254,255].

Recent innovations demonstrate substantial progress. Polyacrylamide/sodium alginate/polypyrrole (PAAM-SA-PPy) hydrogels achieved strain sensitivities of 4.53 over a 400–800% strain range with excellent cyclic stability, attributed to uniformly distributed polypyrrole nanoparticles forming robust 3D conductive networks [251]. Advanced systems incorporating supramolecular interactions—including hydrogen bonding and cation-π interactions—coupled with high-strength polymers achieved tensile strengths of 1.63 MPa, electrical conductivities of 2.16 S/m, and impressive gauge factors of 4.1 (as show in Figure 10II) [256]. Systems enhanced with silver-functionalized carbon nanotubes (Ag-CNTs) within oxidized sodium alginate-polyacrylic acid matrices exhibited exceptional properties: 1412% stretchability, 99.9% antibacterial efficacy, gauge factors of 1.22, and autonomous self-healing through reversible C=N bonds [257]. Additionally, ionic liquid-enhanced polyacrylamide/sodium alginate hydrogels containing graphite fillers achieved ionic conductivities of 22.39 mS/cm^1^, tensile strains of 467%, gauge factors of 4.01, and rapid response/recovery times maintained across over 200 loading–unloading cycles [258].

**Table 7:** Summary of quantitative performance metrics for recent representative alginate-based hydrogels used in strain sensing.

Hydrogel System	Tensile Strength (MPa)	Strain Sensitivity (Gauge Factor)	Stretchability (%)	Ionic Conductivity (S/m)	Response Time (ms)	Recovery Time (ms)	Ref.
PAM-SA-PPy	<1.0	4.53 (400–800% strain)	>800	Conductive	Rapid	Stable (200+ cycles)	[251]
PAM-ALG-PPy	1.63	4.1	Variable	2.16	Optimized	Excellent	[256]
SA/PAM/Ca^2+^/PANI	0.577	3.82	1991	16.51 (25 °C)/11.08 (−20 °C)	High	Dual-temp capable	[259]
OSA/PAA with Ag-CNTs	0.2	Exceptional	1596	Enhanced	0.95	0.86	[260]
Ionic Liquid-PAM/SA	≈0.074	2.081/4.01	467%	≈223.9	186	227	[258]
HCS-SA-Ca^2+^	≈0.281 ± 0.008	Not traditionally calculated	160%	9.80	Immediate response	good	[252]
PBS/STA/SR	4.15 ± 0.21	2.05/2.44/2.61	1531	17.01	17,350	good	[261]
PVA/SA with Dopamine	3.14	High sensitivity	442	Variable	Real-time capable	Flexible to −42.21 °C	[262]

**Table 8:** Comparison of functional and design features for recent representative alginate-based hydrogels used in strain sensing.

Hydrogel System	Primary Application(s)	Key Design Feature	Mechanical Enhancement Strategy	Environmental Stability	Major Limitation	Ref.
PAM-SA-PPy	Strain sensing (human motion)	Uniformly distributed PPy nanoparticles	3D conductive network formation	Standard conditions	Component concentration dependency	[251]
PAM-ALG-PPy	Multi-functional sensing	Hydrogen bonding + cation-π interactions	Hybrid high-strength polymer approach	Improved	Limited long-term immersion data	[256]
SA/PAM/Ca^2+^/PANI	Strain sensing + supercapacitor	In situ aniline polymerization	Multi-network architecture	Dual-temperature	Potential PANI content variation	[259]
OSA/PAA/Ag-CNTs	Wearable strain sensors	Dual crosslinking strategy	Silver-functionalized CNT integration	Excellent	Scalability challenges	[260]
Ionic Liquid-PAM/SA	Strain sensors and flexible electronics	Graphite filler incorporation	Hybrid electrolyte enhancement	Good	Environmental instability under immersion	[258]
HCS-SA-Ca^2+^	Wearable epidermal strain sensors	Dual physical crosslinking network	Double network synergy; Ca^2+^ sustained-release; 3. High amylose starch	Fully biodegradable and non-toxic	Excessive Ca^2+^ loading leads to particle aggregation and structural irregularity; Limited to moderate strain applications	[252]
PBS/STA/SR	Wearable epidermal strain sensors; Underwater communication and distress signaling	Interlocking dual-network structure;. STA bridging layer for enhanced hydrogel-silicone interface bonding	Borax-initiated reversible pH-responsive borate ester bonds with PVA/SA blend; Freeze–thaw crystallization of PVA provides dual-network formation	Expansion rate after 7 days in DI water: 1.00 ± 0.21%; Weight loss over 36 h in air (40% humidity): 38.67 ± 0.38%	Response/Recovery time relatively long	[261]
PVA/SA/Dopamine	Subzero temperature monitoring	Ferric chloride pH regulation	Mechanical interlocking coating	Exceptional (−42.21 °C)	Limited to specific applications	[262]

#### 3.3.2. Environmental Stability and Multifunctional Integration

Environmental stability remains a critical challenge for hydrogel-based sensors, particularly regarding dehydration in atmospheric conditions and swelling in aqueous environments [261,262,263]. Silicone rubber-coated sodium alginate hydrogel fibers with mechanical interlocking structures effectively address these constraints, maintaining stable performance across diverse conditions. pH-regulated ferric chloride-soaked polyvinyl alcohol/sodium alginate dopamine hydrogels achieve superior performance metrics—tensile strengths of 3.14 MPa, elongation of 442%, and operational flexibility down to −42.21 °C—enabling real-time monitoring of finger bending and joint movements across both normal and subzero temperatures [262].

Strategic integration of bioactive components enhances multifunctional capabilities. Conductive hydrogels incorporating S-nitroso-N-acetyl penicillamine and MXene nanosheets combine continuous nitric oxide release with excellent conductivity, essential for sensitive strain sensing applications [264]. Similarly, shape-reversible starch/alginate hydrogels demonstrate substantial tensile strengths of 281.51 kPa and conductivities of 9.80 S·m^−1^, effectively monitoring both subtle body motions (e.g., speech, coughing) and larger movements. These comparative examples illustrate how material composition directly governs mechanical and functional properties [252,265]. Marchiano et al. developed an edible polydopamine-based alginate hydrogel electrode comprising Ca^2+^-crosslinked alginate (3.5% *w*/*v*) plasticized with glycerol (5% *w*/*v*) and reinforced with polydopamine, silver nanoparticles, and food-grade glucose oxidase. The optimized formulation demonstrated an electroactive surface area of 1.99 ± 0.07 cm^2^, double-layer capacitance of 10.1 ± 0.3 μF, and charge-transfer resistance of 7.7 ± 0.6 kΩ. Configured as a first-generation glucose biosensor, the electrode exhibited linear response from 50 μM to 1.0 mM in USP simulated intestinal fluid (pH 6.8), with a detection limit of 10.4 ± 0.8 μM and apparent Michaelis constant (K_M_) of 0.35 ± 0.08 mM. The biosensor retained ≥95% activity during 20 h continuous operation and 90% activity after 30 days of storage, with negligible interference from physiological species. This edible platform establishes a promising route toward ingestible bioelectronics for non-invasive glucose monitoring and personalized metabolic assessment [266].

Advanced multi-network architectures further enhance performance [259,267]. Zhang et al. leveraged a one-step water–oil phase separation strategy to create in situ self-layering bilayer hydrogels with robust interfacial coupling. The top poly(acrylamide-acrylic acid)-gelatin-alginate (poly(AM-AA)-SA-gelatin) network provides mechanical resilience and environmental durability through reversible hydrogen bonding, while the bottom poly(butyl acrylate-2-hydroxyethyl acrylate)-glycerol-polycaprolactone methacrylic anhydride (poly(BA-HEA)-GPCL-MA) pressure-sensitive adhesive (PSA) layer ensures strong yet reversible adhesion to diverse surfaces. The synergistic bilayer architecture that overcomes the conventional adhesion–sensing trade-off without compromising mechanical performance, superior thermal and freeze stability (retaining over 70% and 69.1% of tensile strain after treatment at 60 °C and −25 °C, respectively), and excellent biocompatibility suitable for skin contact applications. The hydrogel exhibits outstanding mechanical properties including maximum strain of 548%, toughness of 12.77 kJ/m^3^, Young’s modulus of 12.5 kPa, and remarkable water retention with only 11% water loss over 30 days at room temperature, with adhesion strength retention exceeding 87.4% after eight cyclic cycles. However, the relatively low conductivity of the PSA layer (0.00025 S/m) may limit direct sensing in the adhesive layer [268]. This work establishes a transformative design paradigm for bilayer hydrogels, positioning the PAAMG system as a promising platform for adaptive soft robotic systems and next-generation wearable sensor technologies.

Similarly, SA/polyacrylamide/calcium/polyaniline hydrogels synthesized via in situ aniline polymerization exhibit exceptional properties: tensile strengths of 0.577 MPa at 1991% elongation, dual-temperature ionic conductivities of 16.51 S/m (25 °C) and 11.08 S/m (−20 °C), strain sensing gauge factors of 3.82, and simultaneous supercapacitor functionality with specific capacitances of 177.19 mF·cm^−2^ [259]. Oxidized sodium alginate/polyacrylic acid/lithium ion hydrogels demonstrate comparable performance with 1596% elongation, 98.6% self-healing efficiency, rapid response/recovery times, and thermo-responsive transparency switching for smart applications [260]. pH-responsive polyacrylic acid/SA/starch hydrogels display pronounced environmental sensitivity—swelling ratios ranging from 400% (pH 2) to 9200% (pH 12)—with exceptional transparency (77–95%), multi-substrate adhesion of 25.6 kPa, and excellent antimicrobial activity (>97%) [267]. Overall, these multifunctional platforms demonstrate sophisticated capabilities in detecting physiological activities—finger bending, pulse rate, electrocardiography signals, and voice recognition—advancing comprehensive health monitoring systems [250,255,269,270,271].

The rapid development of artificial intelligence has also provided new opportunities for the application of hydrogels in wearable devices [272,273,274]. Li et al. synthesized a poly(AM-co-AA)/carboxymethyl cellulose/OSA/polydopamine-modified carbon nanotube (PCOP) hydrogel featuring a dual dynamic cross-linking system combining reversible Schiff-base bonds with robust hydrogen bonding. The hydrogel demonstrates exceptional mechanical and adhesion properties: fatigue resistance exceeding 10,000 deformation cycles with minimal degradation, wet adhesion of 26 kPa within 1 min under simulated sweat conditions, and 90% adhesion retention after 10 attach–detach cycles. Physiological signal acquisition significantly outperforms commercial electrodes, with ECG signal-to-noise ratio of 28.2 dB and EMG signal-to-noise ratio of 10.7 dB during 24 h monitoring. Integration with machine learning algorithms achieves 98.75% validation accuracy for real-time classification of knee joint flexion angles (30°, 60°, 90°, and 120°), enabling precision kinematic feedback. However, scalability concerns for mass production, long-term degradation pathways under extreme environmental conditions, and computational overhead for real-time machine learning in portable systems need further investigation [272]. This work establishes a transformative material platform for intelligent sports science, positioning the PCOP hydrogel as a foundation for AI-enhanced coaching systems and next-generation athletic performance analysis technologies.

#### 3.3.3. Energy Harvesting and Smart Integration

Alginate-based hydrogels have advanced significantly into active energy harvesting applications, marking a pivotal shift from passive sensing toward self-powered wearable electronics [120,275,276,277]. Flexible single-electrode triboelectric nanogenerators incorporating graphene oxide-enhanced polyvinyl alcohol/sodium alginate composites demonstrate substantial performance—outputs of approximately 495 V, currents of 22 μA, and power densities around 4.2 W·m^−2^—effectively powering 80 LEDs with operational stability maintained across −10 °C to 50 °C and durability exceeding 5000 compression cycles. These metrics establish alginate-based systems as viable alternatives for continuous energy generation in wearable applications [120].

**Figure 11:**
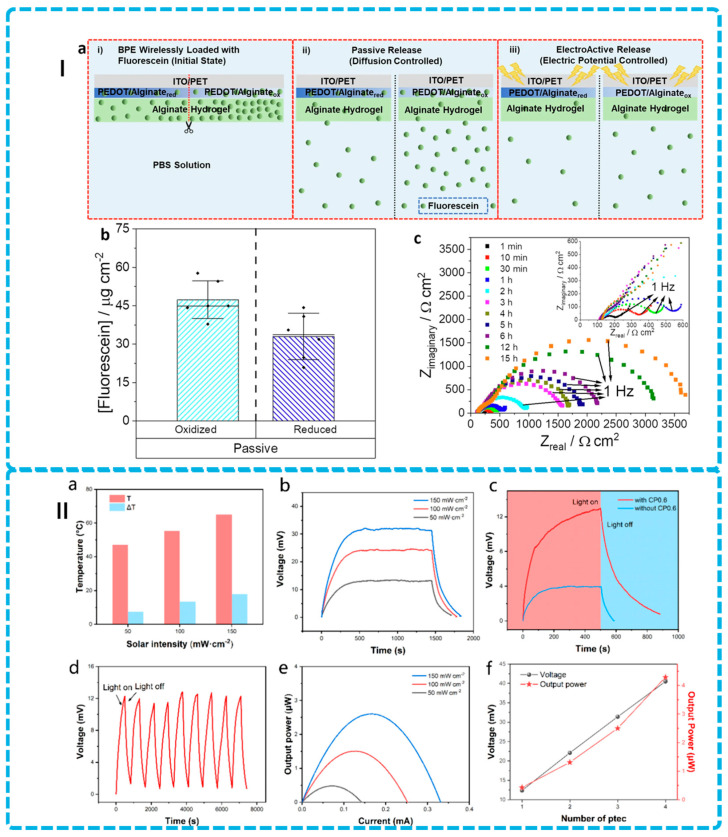
(**I**) Wireless loading of small molecules. (**a**) Schematic representation of the BPE (PET/ITO/PEDOT/Alginate hydrogel) wirelessly loaded with fluorescein molecules (red dashed square), where (**i**) represents the initial state of the intact BPE fully loaded with fluorescein molecules, (**ii**) represents passive (diffusion-controlled), and (**iii**) electrically controlled release from Alginate and PEDOT layers of the BPE halves. (**b**) Concentration of fluorescein passively released from the oxidized (light blue) and reduced (dark blue) halves of the BPE. (**c**) Nyquist EIS plots of a BPE (oxidized half) during passive fluorescein release [278]. (**II**) (**a**) Surface temperature variation in photothermal fabric and temperature differences between the top and bottom of thermoelectric hydrogel under different solar intensities. (**b**) Plot of the voltage output of PTE device under solar intensities. (**c**) Plot of the voltage output of PTE device with CP0.6 and PTE device without CP0.6. (**d**) Cyclic voltage test plots of PTE device under the solar intensity of 50 mW/cm^2^. (**e**) Output power diagram of PTE device under different light intensities. (**f**) Output voltage and output power after connecting multiple PTE devices in series [279].

Complementary hybrid approaches expand energy-harvesting capabilities. Bipolar electrode systems combining polyethylene terephthalate, indium tin oxide, polythiophene, and alginate achieve potential outputs of approximately 350 mV through concentration cell mechanisms, with capacities ranging from 2.7 to 3.3 mAh/g, facilitating both wireless drug delivery and simultaneous energy generation (as shown in Figure 11I) [278]. In addition, Liu et al. addressed the critical limitation of conventional thermoelectric generators in warm climates by developing an integrated photothermoelectric (PTE) device that combines a cotton/polypyrrole (PPy) photothermal layer with a polyacrylamide/sodium alginate thermoelectric hydrogel containing [Fe(CN)_6_]^3−^/^4−^ redox couples, enabling simultaneous harvesting from solar radiation and body heat where body-ambient temperature differentials are thermodynamically inadequate. The synergistic integration of photothermoelectric and thermogalvanic mechanisms sustains stable voltage output of 31.42 mV under 150 mW cm^−2^ solar intensity with maximum Seebeck coefficient of 1.76 mV/K at 2 M LiCl. Single-device power outputs scale linearly with varied solar intensity (as shown in Figure 11II), while four units in series generate 40.5 mV and 4.29 μW, successfully powering LED, calculator, and sensor systems within 120 s via voltage amplifier. However, the single-device power output remains insufficient for standalone applications, necessitating series configuration for practical deployment—a scalability constraint that presents opportunities for modular architecture optimization [279].

Self-powered sensing represents an emerging frontier [255,280,281]. Galvanic copper/polyacrylamide-calcium alginate/silver NO_2_ sensors function independently without external power sources, achieving sensitivities of 307.17% per ppm with detection limits of 2.86 ppb [280]. Advanced human–machine interfaces, such as polyaniline@silk fibroin/carbon nanotube/graphene oxide interpenetrating networks, enable sophisticated functionalities—including voice recognition, comprehensive motion monitoring, and wireless robotic arm control via smart gloves—demonstrating the feasibility of fully integrated wearable systems [255].

**Figure 12:**
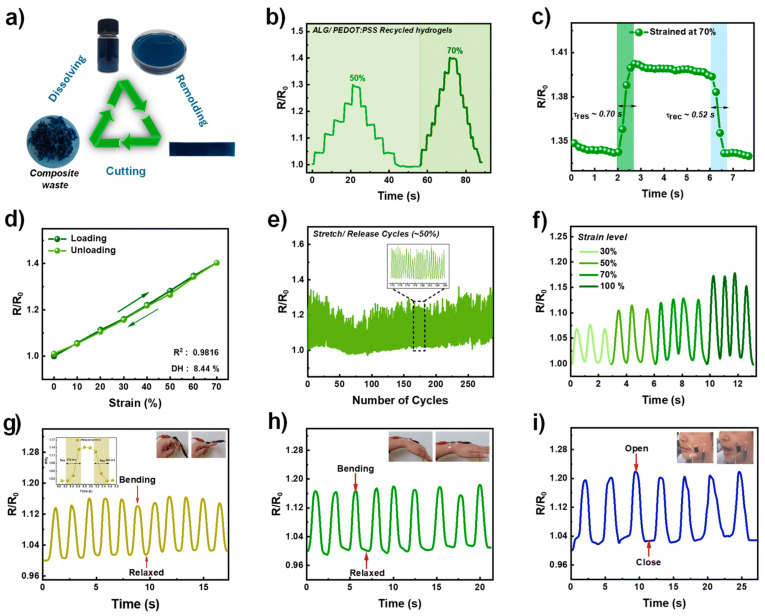
(**a**) Recycling process of hydrogels. (**b**) Relative resistances of the ALG/PEDOT:PSS recycled hydrogel strain sensors under consecutive step-and-hold testing. (**c**) Response and recovery times of the sensor applied under a strain of 70%. (**d**) Sensitivity responses of the ALG/PEDOT:PSS recycled hydrogels during loading and unloading cycles with a GF and linear curves of the strain sensor. (**e**) Resistance changes upon repeated loading and unloading for 300 cycles at a strain of 50%. (**f**) Cyclic stretching–releasing of the ALG/PEDOT:PSS recycled hydrogels at different strain levels. A demonstration of the recycled sensor for (**g**) finger bending, (**h**) wrist bending, and (**i**) mouth opening/closing [282].

Recyclability and sustainability emerge as critical advantages. Glycerol-plasticized alginate/PEDOT:PSS hydrogels demonstrate exceptional properties: 138% stretchability, ultralow hysteresis of 2.95% at 50% strain, and outstanding recyclability maintaining consistent performance over 450 stretching cycles with complete dissolution and reformation capability (as shown in Figure 12) [282]. This regenerative capacity distinguishes alginate-based platforms from conventional non-recyclable energy harvesting materials, positioning them as environmentally sustainable alternatives while maintaining functional reliability across extended operational lifespans.

#### 3.3.4. Sphere-Based Soft Actuators

Alginate-based hydrogels can be shaped into spherical beads whose isotropic geometry and modular architecture enable encapsulation and scalable, stimuli-responsive actuation across diverse thermal, magnetic, electrical, optical, and chemical domains [283]. In comparison to traditional planar or cylindrical hydrogel actuators, spherical alginate structures offer distinct advantages, including facile fabrication via droplet microfluidics or centrifugal casting, the ability to encapsulate active or living agents, and adaptability for anisotropic deformation through directional fillers or asymmetric stimuli-responsive domains [119,283,284,285,286,287,288].

Recent developments aptly illustrate these capabilities. Salahuddin et al. developed biomorph soft actuators (BSAs) that replicate the resilience of tardigrades by combining a thermo-responsive PNIPAm hydrogel core with a porous alginate shell [288]. These hollow spheres exhibit contraction under dehydration or microwave heating and re-expansion upon rehydration, enabling reversible formations and durability under extreme conditions. When embedded in McKibben-style sleeves, BSAs achieved contraction strains exceeding 9% with accelerated microwave responses, showcasing their significant potential for high-speed soft robotic systems and environmental remediation applications, such as removing heavy metals from industrial effluents [288]. Harder et al. developed soft, untethered hydrogel microrobots capable of targeted single-cell delivery, spheroid assembly, photothermal actuation, and real-time thermal sensing [289]. The microrobots consist of alginate hydrogel networks embedded with gold nanorods for plasmonic heating and Rhodamine B for temperature monitoring. Microfluidic encapsulation produces uniform ~30 μm diameter spheres with surface modifications enabling electrostatic cell adhesion and integrin-mediated spheroid formation. Thermophoretic convection driven by 785 nm laser irradiation enables precise locomotion and manipulation in three-dimensional workspaces. The microrobots simultaneously function as localized heaters and sensors, modulating the cellular microenvironment through photothermal actuation while providing real-time temperature feedback. Single-cell capture and spheroid formation are achieved with high efficiency and cell viability >95% over 72 h. Combined photothermal stimulation and chemotherapy demonstrated synergistic reduction in fibrosarcoma cell invasiveness in proof-of-concept studies, establishing the platform as a versatile tool for integrated drug screening and cellular manipulation [289].

#### 3.3.5. Summary

In summary, alginate-based hydrogels have established themselves as transformative materials in flexible electronics and soft robotics, leveraging their unique properties for a wide array of applications, particularly in strain sensing, energy harvesting, and physiological monitoring [264]. Despite significant advancements, challenges including component concentration dependency, performance degradation during prolonged immersion, and environmental instability persist [261]. Continued research into environmental stability and the development of multi-network architectures are crucial for practical applications. Looking forward, integrating advanced nanomaterials and dual-responsive polymers will be essential in advancing alginate-based hydrogels as foundational materials for sustainable wearable electronics and personalized healthcare solutions.

### 3.4. Food Industry Applications: Integrated Detection and Preservation Systems

Alginate-based hydrogels have emerged as versatile multifunctional materials in food safety applications, functioning simultaneously as contaminant detection platforms and removal systems [290,291,292]. Their inherent biocompatibility, combined with abundant hydrophilic functional groups (−OH and −COOH), enables rapid detection of diverse food contaminants—including pesticides, heavy metals, pathogenic microorganisms, and veterinary drugs—while maintaining high recovery rates (>85%) and excellent mechanical stability critical for practical sensor integration. Strategic material design consolidates disparate functional requirements within unified platforms, addressing interrelated challenges spanning therapeutics, environmental remediation, and food industry operations through a cohesive engineering approach [290,291,292,293]. This section systematically examines the latest developments, functional capabilities, and technical challenges associated with alginate-based hydrogels in food safety applications across production, processing, and distribution stages. Quantitative performance metrics and mechanistic and practical features for recent representative alginate-based hydrogels used in food detection systems are summarized in Table 9 and Table 10, while quantitative performance metrics for recent representative alginate-based hydrogels used in food preservation and packaging systems are summarized in Table 11.

#### 3.4.1. Pesticide Detection

Pesticides are essential agricultural inputs yet they pose significant ecological and food safety concerns through bioaccumulation in food chains and tissue accumulation [303,304]. Pesticide residues persist as a widespread contamination challenge, particularly in foods of animal origin—including meat, fish, eggs, and milk [290,305]. Conventional detection methods face inherent limitations in sensitivity, multiplexing capability, and operational practicality. Alginate-based hydrogel sensors overcome these constraints through multimechanistic detection platforms, demonstrating exceptional analytical performance for pesticide monitoring [294,306,307].

Chen et al. pioneered a target-responsive system by embedding phosphotriesterase hybrid nanoflowers within sodium alginate hydrogels to detect methyl parathion. This biocatalytic approach exploits enzymatic degradation of the target pesticide to p-nitrophenol, generating colorimetric signals quantifiable via smartphone imaging without requiring sophisticated instrumentation. The system achieved a broad linear detection range (1–150 μM), low detection limit (0.15 μM), and excellent long-term stability, establishing enzyme-integrated hydrogels as viable point-of-care diagnostic platforms [294].

Building on enzymatic principles, Wang et al. advanced detection capabilities through dual-signal amplification using bifunctional gold nanoenzymes integrated within sodium alginate hydrogels, achieving detection limits of 7 ppb with response times under 25 min [295]. Complementary to enzymatic detection, Qi et al. developed a plasmonic sensing platform by incorporating gold–silver core–shell nanoparticles within sodium alginate frameworks. The hydrogel’s inherent porosity creates consistent surface-enhanced Raman spectroscopy (SERS) hotspots, enabling label-free thiram residue detection in fruit juice at concentrations as low as 1 × 10^−10^ M without requiring complex sample preparation. This approach demonstrates the versatility of alginate platforms in accommodating diverse physicochemical detection mechanisms while maintaining practical applicability [306].

#### 3.4.2. Heavy Metal Detection and Removal: Dual-Functionality Systems

Industrial activities release hazardous heavy metals into aquatic and terrestrial ecosystems, creating persistent bioaccumulation risks that threaten food chain integrity and human health. Alginate-based hydrogels uniquely integrate detection and removal capabilities within single-platform systems, addressing this dual challenge through sophisticated material design [297,308,309].

For instance, Li et al. developed a sodium alginate-based fluorescent hydrogel achieving exceptional analytical performance for Cr(VI) monitoring, with a detection limit of 1.99 nM and recovery rates of 100–108% while maintaining specificity against 22 interfering ions. This performance validates alginate’s potential for food contact safety applications requiring high selectivity and sensitivity [296].

Complementary to detection, Tang et al. advanced removal efficacy through chitosan/sodium alginate/calcium hydrogels, achieving a tensile strength of 0.190 MPa and swelling capacity of 89 g/g at pH 3. These properties facilitate rapid analyte penetration into internal pores, enabling effective sequestration of lead, copper, and cadmium ions through multiple binding mechanisms [310].

**Figure 13:**
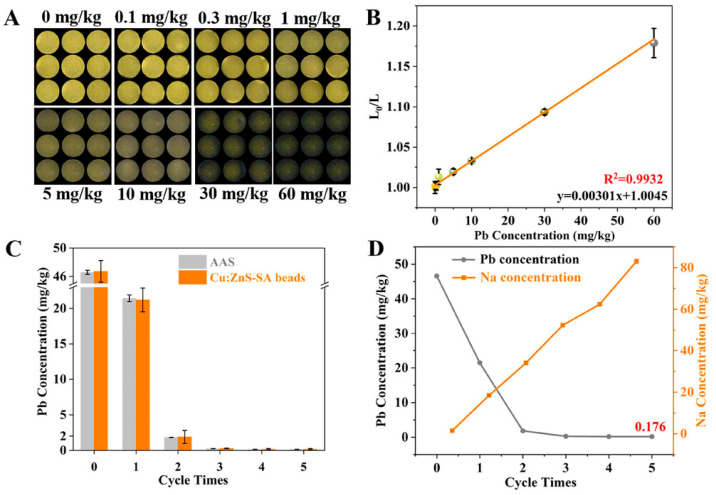
(**A**) Change in fluorescence brightness of hydrogel beads after reacting with 0, 0.1, 0.3, 1, 5, 10, 30, 60 mg/kg Pb(II); (**B**) fitting curve of the brightness ratio “L_0_/L” and Pb(II) concentration; (**C**) results of Pb(II) determined by hydrogel beads were compared with results of standard AAS method; (**D**) concentration changes in Pb(II) and Na(II) during the continuous cycles. AAS, atomic absorption spectroscopy; SA, sodium alginate [297].

Furthermore, Lan et al. developed a bifunctional Cu:ZnS quantum dot–sodium alginate (Cu:ZnS QDs-SA) hydrogel demonstrating simultaneous visual detection and efficient removal of lead in liquid food matrices. The system employs aggregation-induced quenching (ACQ) for label-free detection across a linear range of 0.1–60 mg/kg, achieving a quantification limit of 0.1 mg/kg (as shown in Figure 13) [297]. Integration with an automated cyclic device yielded outstanding Pb^2+^ removal efficiency of 99.62% across diverse beverage matrices—including lake water, herbal tea, orange juice, and Chinese baijiu—demonstrating practical applicability across complex food systems. The dual functionality originates from synergistic material design: Cu:ZnS quantum dots enable fluorescence-based detection through quenching mechanisms, while abundant sodium alginate functional groups facilitate chemisorption via complexation and ion exchange pathways. Toxicity assessment revealed minimal cytotoxicity (cell viability > 87% under extreme conditions), confirming biocompatibility for environmental and food safety applications. However, current limitations restrict practical deployment; the Cu:ZnS QDs-SA system’s applicability remains confined to liquid food matrices, and field-scale validation in complex food production environments remains incomplete [297].

#### 3.4.3. Veterinary Drug and Antibiotic Detection

The widespread application of veterinary drugs and antibiotics in animal husbandry generates persistent environmental contamination and accelerates antimicrobial resistance through selective pressure on microbial populations—a critical global health challenge [311,312]. Alginate-based hydrogel platforms address this challenge through diverse sensing mechanisms tailored to specific antibiotic classes.

Jie et al. pioneered europium-functionalized porphyrin-based metal–organic framework heterostructures (MOF@Eu) integrated within polyvinyl alcohol hydrogels, achieving rapid sulfonamide detection at 56 nmol/L with high recovery rates (81.2–118.3%). This approach leverages the hydrogel’s inherent microporosity and water permeability to facilitate analyte transport while the MOF structure provides selective binding sites [313].

Complementing MOF-based approaches, Mei et al. developed lutetium-doped cadmium telluride quantum dot (Lu^3+^/CdTe QDs) hydrogel sensors achieving exceptional response times (≤2 s) for doxycycline detection at 36.6 nM with recovery rates of 94–103%. The rapid response originates from optimized pore architecture facilitating direct analyte penetration into the quantum dot sensing layer [314].

Advancing detection sophistication, Ao et al. developed hydroxyl-functionalized covalent organic frameworks (COFs) integrated with sodium alginate/calcium/polyacrylate microspheres, enabling complete fluoroquinolone separation within one minute without centrifugation [315]. Combined with HPLC-MS/MS validation, this platform achieved outstanding analytical performance: broad linear detection range (0.01–100 ng/mL), low detection limits (0.05–0.42 μg/kg), high precision (RSD ≤ 20.5%), and excellent recovery rates (80.3–117.3%) across diverse food samples. Critically, extraction efficiencies exceeded 90% across five consecutive cycles, demonstrating practical reusability and cost-effectiveness [315].

These integrated systems collectively demonstrate how synergistic material design—combining selective binding frameworks (MOFs, COFs) with transport-optimized hydrogel matrices—enables simultaneous achievement of sensitivity, speed, and practical operability required for food supply chain monitoring.

#### 3.4.4. Pathogen Detection: Advanced Biosensing for Foodborne Disease Prevention

Foodborne pathogens—including viruses, bacteria, fungi, and parasites—represent significant global health threats. Five bacterial species collectively account for 54.9% of pathogen-related deaths, with the U.S. Centers for Disease Control and Prevention reporting approximately 48 million annual foodborne illnesses, 128,000 hospitalizations, and 3000 deaths [316]. This epidemiological burden underscores the critical need for sensitive, rapid detection systems. Alginate-based hydrogels address this challenge through integration with molecular recognition technologies, particularly aptamer and CRISPR methodologies [298,317].

Zhang et al. pioneered aptamer-functionalized carbon dot integration within hydrogel matrices, enabling simultaneous detection of *E. coli* O157:H7 (8 CFU/mL) and *S. aureus* (20 CFU/mL) with exceptional recovery rates (90–101%) across complex food samples [317]. Advancing this approach, Yu et al. developed a dual-mode smart hydrogel aptasensor for *Vibrio parahaemolyticus* quantification, combining visual fluorescence with microfluidic analysis to achieve rapid screening at 100 CFU/mL (45 min) and accurate quantification at 10 CFU/mL (45–60 min) through ATP aptamer-gold nanocluster signal amplification [318].

Complementary to single-target approaches, Xu et al. developed a phage/aptamer dual-encoded alginate hydrogel microfluidic platform enabling simultaneous detection of *E. coli*, *Salmonella typhimurium*, and *Staphylococcus aureus* within 60 min at exceptionally low limits (20, 30, and 15 CFU/mL, respectively) [298]. This represents a significant advancement toward high-throughput pathogen screening. Further innovation through CRISPR/Cas12a biosensing, demonstrated by Shen et al., exploited alginate’s sol–gel transition properties for *Salmonella* detection, achieving 158 CFU/mL sensitivity with outstanding recovery rates (87.91–98.63%) through precise enzymatic control via CRISPR-triggered alkaline phosphatase release and modulated water molecule diffusion [299].

These integrated platforms collectively demonstrate how rational design combining molecular recognition elements (aptamers, CRISPR), stimuli-responsive hydrogel matrices, and microfluidic integration enables simultaneous advancement of sensitivity, multiplexing capability, and operational speed—essential requirements for real-time food supply chain monitoring.

#### 3.4.5. Smart Food Packaging and Preservation: Active Preservation Systems

Food packaging serves dual critical functions—preserving product integrity while ensuring safety throughout distribution chains. Biodegradable alternatives to conventional petroleum-based materials address mounting sustainability concerns [319]. Alginate-based hydrogels have emerged as advanced materials for intelligent multifunctional packaging, distinguished by their capacity to integrate antimicrobial and antioxidant functionalities within structurally stable matrices [291,301,320,321].

Soy protein isolate/sodium alginate composite films incorporated with ε-polylysine and tannic acid exemplify this functional integration, demonstrating enhanced mechanical performance (increased tensile strength and elongation), substantial radical-scavenging capacity (78% DPPH activity), and measurable preservation efficacy—extending refrigerated beef shelf life by three days through synergistic antimicrobial mechanisms [322].

Building upon these principles, Abdin et al. developed sophisticated biodegradable films combining carboxymethyl cellulose, sodium alginate, and Thymus vulgaris leaf extract at controlled concentrations. Systematic variation in extract concentration yielded progressive performance enhancements: increased opacity (2.98), while simultaneously reducing moisture content (10.31%), swelling capacity (30.17%), and water vapor permeability (1.12 × 10^−10^ g·m^−1^·s^−1^·Pa^−1^) with morphological transition from smooth to irregular surface topography. This rational composition design effectively preserved moisture content, titratable acidity, and sensory attributes of packaged cheddar cheese during cold storage, demonstrating clear performance superiority over commercial packaging alternatives [323].

These integrated systems illustrate how strategic material design—combining natural antimicrobial/antioxidant components with transport-optimized hydrogel matrices—simultaneously achieves mechanical stability, barrier functionality, and extended preservation efficacy required for diverse food preservation applications.

#### 3.4.6. Food Freshness Detection

Rapid, practical, and reliable freshness assessment represents a critical requirement for food safety throughout distribution chains [324,325]. Intelligent alginate-based hydrogel indicators with stimulus-responsive properties have emerged as superior alternatives to conventional time–temperature indicators, enabling real-time freshness monitoring with visual or fluorescent readouts [300,326,327].

For instance, Qiao et al. pioneered a novel approach by developing a glycerol-sodium chloride antifreeze organic hydrogel (GSC-AOH) incorporating glucono-δ-lactone and a methyl red/bromothymol blue dual-indicator system to assess rice storage duration. This thermos-responsive platform maintains structural integrity across extreme temperature ranges (−25 °C to 25 °C), achieving rapid detection times (1.17 min at 25 °C; 1.36 min at −25 °C) with exceptional selectivity for free fatty acids accumulated during storage. The hydrogel’s innovative color gradient progression—transitioning from green (<1 year) to orange (>3 years)—enables quantitative assessment of storage duration, effectively bridging a critical gap where conventional hydrogels fail under extreme cold conditions [326].

Complementing temperature-responsive approaches, Li et al. developed a self-ratiometric fluorescent sensor by embedding a synthesized coumarin derivative (DCCA: 7-diethylamino-coumarin-3-carboxylic acid) within sodium alginate hydrogel beads via calcium chloride crosslinking [300]. This system achieves remarkable analytical performance through dual fluorescence emission with an exceptional ratiometric fluorescence ratio change (I_480_/I_630_) of ~500× across pH 2.5–7.4, demonstrating excellent linearity in highly acidic ranges (pH 2.5–5.0, R^2^ = 0.9846). Enhanced biocompatibility (>85% cell viability), resistance to 25 interfering metal cations/anions, and rapid response kinetics enable RGB data conversion for precise quantitative pH determination. This represents the first successful visual detection of extremely acidic pH changes via self-ratiometric fluorescence in alginate hydrogels, enabling real-time fruit juice freshness monitoring with outstanding recovery rates (98–102%) across diverse acidic matrices—including orange, prickly pear, and lemon juices—while maintaining structural stability exceeding four weeks at 4 °C [300]. However, practical limitations restrict current deployment: restriction to highly acidic pH ranges (2.5–5.0), UV light exposure requirements (<2 min to prevent photobleaching), and validation limited to acidic food systems necessitate further development for broader food industry applicability.

Furthermore, Georgopoulou et al. developed the first fully edible thermoelectric–electrochromic device that harvests thermal energy from food to generate electrical power with integrated visual feedback. The system combines vanillin-crosslinked chitosan (p-type) and calcium-crosslinked alginate (n-type) hydrogels functionalized with food-grade potassium chloride electrolyte. Anthocyanin-functionalized gelatin electrochromic displays reversibly shift from purple to blue under 1 V, enabling visual temperature monitoring that completes within 10–15 min as food cools to safe consumption temperature. Unlike previous ionic thermoelectric hydrogels, this platform operates under modest temperature gradients without requiring non-edible components. Despite modest power output compared to conventional thermoelectric devices, the system shows promise for on-food temperature monitoring with safety indicators signaling optimal consumption timing and for integration into smart food packaging [102]. This work establishes a foundation for sustainable, biodegradable edible electronics, demonstrating that food-grade materials can be engineered to harvest thermal energy and provide actionable visual signals for food science, biomedical monitoring, and transient electronic applications.

#### 3.4.7. Summary

Alginate-based hydrogels enable rapid (<10 min), sensitive detection with >85% recovery in food safety applications [290,294,295,328]. These biodegradable materials are poised to serve as next-generation intelligent food safety systems throughout production (pesticide and veterinary drug detection), processing (pathogen detection and removal), and distribution (active packaging) [290,291,292]. Despite the promising capabilities demonstrated in detecting pesticides, heavy metals, and pathogenic bacteria, challenges such as specificity, the need for direct food contact, susceptibility to UV photobleaching, and the necessity of field-scale studies remain [324,325]. Overall, alginate-based hydrogels are positioned as practical food safety solutions that advance preservation strategies essential for protecting public health.

### 3.5. Other Emerging Applications

#### 3.5.1. Enhanced Flame Retardancy in Alginate-Based Hydrogels

Alginate-based hydrogels have emerged as effective flame-retardant materials for protective textiles and equipment, benefiting from their flexible structure and high water content, which facilitate evaporation-driven thermal energy absorption [329,330,331,332,333,334]. For instance, Yu et al. demonstrated that alginate–cotton fabric composites, when incorporated with calcium chloride (CaCl_2_), could withstand temperatures exceeding 1200 °C for 30 min without igniting the underlying fabric, whereas untreated cotton ignited in just 12 s [330]. This remarkable performance is attributed to significant alterations in thermal degradation mechanisms—specifically, the incorporation of Ca^2+^ ions shifts the degradation pathway of alginate, promoting decarboxylation and esterification over char residue formation, thereby minimizing flammable gas production and delaying ignition [332,333].

Further advancements in flame resistance are evident in composite hydrogels. Xiao et al. developed sodium alginate-carboxymethyl cellulose-N-isopropylacrylamide composites that achieved an ignition time of 79 s, a heat release rate of 113.1 kW·m^−2^, and a total heat release of only 29.3 MJ/m [333]. Layer-by-layer assembly techniques, exemplified by Wang et al.’s sodium alginate-chitosan coating on polyurethane foam, significantly reduced peak heat release, total heat release, and smoke production by 66%, 11%, and 6%, respectively, with minimal weight addition (5.7%) [334]. Collectively, these developments position hydrogel-based systems as scalable and effective flame-retardant solutions for protective applications, addressing critical safety needs in various environments.

Nonetheless, challenges remain in achieving uniform distribution of alginate complexes within host materials. Inadequate dispersion may result in inconsistent flame retardancy and inadequate protection, undermining overall effectiveness and restricting broader practical applications [335]. Addressing this inconsistency is essential for enhancing the reliability of these flame-retardant systems.

#### 3.5.2. Electromagnetic Interference (EMI) Shielding

The rapid expansion of fifth-generation (5G) communication technologies and electronic devices has elevated living standards while intensifying concerns regarding electromagnetic interference (EMI) and radiation hazards [336]. Prolonged exposure to high-intensity electromagnetic fields poses significant health risks, particularly to sensitive medical implants and precision instruments [337]. Consequently, there is an increasing demand for effective electromagnetic shielding materials capable of mitigating these risks [337,338,339].

Alginate-based hydrogels have demonstrated substantial potential for absorbing and shielding electromagnetic waves (EMWs), owing to their unique porous structure and specific functional modifications. Recent studies exemplify this approach [336,337,338]. For instance, CPSA hydrogels, composed of carbon nanotube (CNT)-wrapped polyacrylamide (PAM) particles integrated with sodium alginate and crosslinked with Cu^2+^, exhibited an evaporation rate of 1.97 kg·m^−2^·h^−1^ under 1.0 sun irradiation and provided an electromagnetic shielding effectiveness of 81.1 dB at a thickness of 6 mm, largely through absorption mechanisms [338]. This dual functionality also encompasses excellent photocatalytic activity, as evidenced by methylene blue degradation in wastewater treatment.

In another advancement, He et al. designed multi-layered alginate hydrogels with alternating layers of cobalt-carbon nanotubes (Co-CNT) and standard CNTs. The introduction of cobalt enhances electromagnetic wave attenuation, minimizes secondary reflection, and improves impedance matching for effective wave penetration. A 0.7 mm thick multi-layered structure demonstrated over 99.8% attenuation of electromagnetic waves, achieving absorption losses greater than 85% [339]. These studies collectively underscore the potential of absorption-dominated, flexible alginate hydrogels in addressing emergent challenges in EMI shielding, facilitating integrated solutions for water-energy management.

#### 3.5.3. Soil Conditioning and Agricultural Applications

Soil stabilization is essential for overcoming the difficulties associated with silty soils, which often exhibit low shear strength, poor consolidation, and dynamic instability. Conventional engineering techniques frequently involve extended curing periods of 28 to 90 days, significantly contributing to CO_2_ emissions, which account for approximately 8% of global carbon output [300]. However, recent innovations are fostering more efficient soil stabilization methods. For instance, Yin et al. developed a ternary interpenetrating polymer network consisting of sodium alginate, acrylamide, and hexadecyl methacrylate, which achieved an unconfined compressive strength of 2405.69 kPa, nearly 25 times higher than untreated soil, while maintaining a hydraulic conductivity of 3.45 × 10^−6^ cm/s. This advancement effectively reduces the need for protracted curing times, thus improving construction efficiency [300].

Additionally, waste valorization initiatives are converting construction and demolition debris into high-performance materials. Magombana et al. developed calcium-alginate hydrogel amendments using crushed construction waste to enhance the mechanical properties and ductility of soils, making them suitable for pavement base courses [301]. To combat the environmental challenges associated with petroleum-based hydrogels, He et al. introduced a biodegradable lignin-based ternary composite containing 7.00% nitrogen through Mannich reaction chemistry. These composite exhibits remarkable water absorption capacity of 91.9 g/g and recyclability of 88.95 g/g after eight cycles [302].

Significantly, within just one month of application, this composite demonstrated a 27.88% increase in water holding capacity, a 42.66% improvement in water retention, and an extended nutrient release profile 48 to 60 times longer than conventional fertilizers. Furthermore, soil organic matter increased by 222.40%, while total nitrogen content rose by 110.05%, indicating substantial improvements in soil fertility due to increased microbial activity [302]. These advancements highlight the efficacy of alginate-based materials and innovative composites in enhancing soil stabilization and agricultural practices, thereby advancing sustainable construction methods and promoting soil health.

## 4. Conclusions and Perspectives

Alginate-based hydrogels are versatile biomaterials whose properties are controlled by molecular structure (M/G balance), crosslinking strategy (physical/chemical), and compositional modifications. This enables tailored performance across biomedical and environmental applications [2,6,20,29,340,341,342]. Current clinical applications in wound dressings, drug delivery, and tissue engineering scaffolds confirm that alginate-based materials can successfully translate from laboratory research into practical therapeutic solutions [209]. Nevertheless, important challenges remain: achieving robust mechanical properties without sacrificing biocompatibility, controlling degradation kinetics predictably, and navigating the complex regulatory and manufacturing landscape required for practical approval [16,22,209,343,344,345].

To facilitate rational selection of preparation strategies for specific applications, we synthesize our comprehensive review findings in Table 12. This matrix systematically integrates preparation-application relationships across biomedical, environmental, and technological domains, providing actionable guidance for researchers, engineers, and practitioners.

Ionic crosslinking demonstrates exceptional suitability for cell encapsulation and injectable delivery applications, leveraging rapid gelation kinetics under physiological conditions, superior biocompatibility, and outstanding scalability—characteristics that render these systems particularly valuable for minimally invasive therapeutic delivery, personalized medicine approaches, and cost-sensitive manufacturing environments [15,31,34,56,58,59,60,61,62,67,68,69]. However, modest mechanical properties (typically <10 MPa) and unpredictable degradation kinetics driven by ion-exchange mechanisms limit broader applicability to non-load-bearing contexts.

Conversely, covalent crosslinking strategies deliver superior mechanical robustness (9–110 MPa tunable) and prolonged structural stability essential for load-bearing tissue scaffolds, regenerative medicine applications, and long-term implant functionality; however, photo-initiator cytotoxicity—particularly from VA-086, VA-044, Irgacure-1870, and related photo-initiators—presents significant biocompatibility challenges requiring careful mitigation through alternative photo-initiator development, sequential crosslinking strategies, or visible-light activation methodologies [34,108,109,111,112].

Hybrid approaches leveraging complementary mechanisms—such as sequential ionic and photochemical crosslinking (e.g., GelMA/Oxidized SA systems achieving >95% cell viability) or Schiff base chemistry combined with metal coordination—effectively circumvent individual limitations while enabling synergistic functional enhancement, positioning these systems as optimal solutions for emerging complex applications including 3D/4D bioprinting, stimuli-responsive therapeutics, dynamic mechanical response, and multifunctional tissue engineering scaffolds [3,118,183,217,260,340].

Physical crosslinking systems maximize cost-effectiveness and manufacturing simplicity while maintaining exceptional biocompatibility and reversible gel properties; however, inherent mechanical limitations restrict applicability to dynamic, reversible systems and non-load-bearing applications such as responsive drug delivery and sensor integration [78,79,80,81,82].

Strategic selection of appropriate preparation methodologies requires systematic consideration of interconnected parameters: mechanical property requirements aligned with specific tissue targets, degradation timeline synchronized with biological healing kinetics, biocompatibility constraints for cellular interactions, scalability demands for clinical translation, and regulatory pathway complexity for commercial deployment. This integrated decision framework, synthesized in Table 12, enables researchers to navigate the extensive alginate preparation landscape with precision, maximizing probability of successful translation toward clinical and commercial endpoints while maintaining rigorous safety and efficacy standards.

The path forward requires addressing these gaps while capitalizing on emerging technologies to unlock new possibilities for alginate systems [113,209,346,347,348,349,350]. Stimuli-responsive hydrogels that respond intelligently to physiological signals—enabling triggered drug release, real-time wound monitoring, and tissue-guided regeneration—represent an exciting frontier worthy of sustained exploration [2,7,351,352]. Machine learning and artificial intelligence offer powerful tools for accelerating hydrogel design optimization, while high-throughput screening can rapidly evaluate formulations for specific applications [7,29,272]. Complementary advances in fabrication technologies—particularly 3D/4D bioprinting, microfluidics, and electrospinning—promise construction of complex tissue-like architectures with unprecedented precision [3,289,340,353]. Equally important is the shift toward sustainable manufacturing: embracing circular economy principles, exploring alternative alginate sources through bacterial synthesis and waste valorization, and developing controlled degradation strategies synchronized with biological healing timelines [2,26]. Establishing standardized characterization protocols would also facilitate cross-study comparison and expedite regulatory pathways [3,340].

Translating these advances into clinical reality demands genuine collaboration across disciplines—materials scientists, biomedical engineers, clinicians, and regulatory experts must work together seamlessly [354]. Rigorous clinical validation studies, comprehensive biocompatibility assessments, and GMP-compliant manufacturing represent non-negotiable requirements for regulatory approval [2,8,22,209,340,345]. Partnerships between academic research groups, industry, and regulatory bodies will be essential for streamlining this process while maintaining rigorous safety standards [354]. Alginate-based hydrogels show promise in environmental remediation, agriculture, and bioelectronics [209]. Interdisciplinary collaboration integrating molecular understanding, advanced fabrication, and clinical validation will establish these materials as cornerstone biomaterials for modern medicine and technology.

## Figures and Tables

**Table 2:** Comprehensive crosslinking strategy comparison.

Performance Parameter	Ionic Crosslinking	Physical (Entanglement)-Based	Chemical (Covalent) Crosslinking	Hybrid Crosslinking
Swelling Ratio/%	1500–4200 (pH 7.4, 37 °C, 24–72 h)	400–9200 (pH-dependent; freeze–thaw cycled)	60–400 (pH 7.4, 37 °C, 24 h)	800–2000 (pH 5.0–7.4, 37 °C, 48 h)
Water Content (%)	74–94% (gravimetric analysis)	78–96%	40–75% (high crosslinking density)	65–85% (intermediate)
Tensile Strength (MPa)	0.01–0.5	0.1–1.5	5–110	1–15
Fatigue Durability/cycles (Loading Cycles to 50% Strength Retention)	50–200	500–2000	>10,000	2000–8000
Compressive Strength (kPa)	10–100	50–250	500–5000	200–1000
28-Day Weight Retention (%)	40–70%	60–85%	85–98%	75–90%
Cell Viability (LIVE/DEAD staining, 72 h post-encapsulation) (%)	>90%	85–95%	60–85%	80–95%
Gelation Time (seconds)	5–60 s	5–30 min	2–20 min	10–120 s
Manufacturing Cost (USD/g material)	$0.50–2.00	$1.00–3.00	$2.00–5.00	$2.50–6.00
Primary Applications	Drug delivery, cell encapsulation, soft tissue engineering	Bioprinting, regenerative medicine scaffolds	Load-bearing tissue engineering, flexible electronics	Multi-functional devices requiring mechanical adequacy + biocompatibility

**Table 9:** Summary of quantitative performance metrics for recent representative alginate-based hydrogels used in food industry applications—detection systems.

Hydrogel System	Target Analyte	Detection Limit	Linear Range	Recovery Rate (%)	Response Time	Ref.
SA/Phosphotriesterase Nanoflowers	Methyl parathion	0.15 μM	1–150 μM	>90	3 min	[294]
SA with Au-Nanoenzymes	Pesticides	7 ppb	Broad	>90	<25 min	[295]
SA Fluorescent Hydrogel	Cr(VI)	1.99 nM	Linear range confirmed	100–108	Rapid	[296]
Cu:ZnS QDs/SA	Pb(II)	0.1 mg/kg	0.1–60 mg/kg	99.62 (removal)	Real-time	[297]
Phage/Aptamer Dual-Encoded SA	*E. coli*/*S. typhimurium*/*S. aureus*	20/30/15 CFU/mL	Species-specific	>85	60 min	[298]
CRISPR/Cas12a/SA	Salmonella	158 CFU/mL	Concentration-dependent	87.91–98.63	Rapid	[299]
DCCA/SA Beads	Acidic pH monitoring	pH 2.5–7.4 range	pH 2.5–5.0 (R^2^ = 0.9846)	98–102	Real-time	[300]

**Table 10:** Comparison of mechanistic and practical features for recent representative alginate-based hydrogels used in food industry applications—detection systems.

Hydrogel System	Detection Mechanism	Readout Method	Food Contact Safety	Field Applicability	Major Advantage	Limitation	Ref.
SA/Phosphotriesterase	Enzymatic degradation → colorimetric	Smartphone imaging	Confirmed	Point-of-care kit	High enzyme stability	Limited to specific pesticides	[294]
SA/Au-Nanoenzymes	Dual-signal amplification	Visual + instrumental	Tested	Portable	Multiplexing capability	Complex matrix interference	[295]
SA Fluorescent (Cr detection)	Fluorescence quenching	Fluorometric	Validated	Laboratory	Specificity vs. 22 interfering ions	Requires fluorescence setup	[296]
Cu:ZnS QDs/SA	Aggregation-caused quenching (ACQ)	Visual + automated cyclic device	Validated in multiple beverages	Automated integrated system	Dual functionality (detection + removal)	Complexity of system design	[297]
Phage/Aptamer/SA	Biological capture + microfluidic separation	Electrophoresis chip analysis	Confirmed	Microfluidic platform	Simultaneous multi-pathogen detection	Complex preparation	[298]
CRISPR/Cas12a/SA	Sol–gel transition triggered release	Magnetic relaxation switching	Emerging	Laboratory-based	CRISPR integration	Requires NMR analyzer	[299]
DCCA/SA	Self-ratiometric fluorescence	RGB data conversion	High biocompatibility (>85%)	Real-time juice monitoring	First visual self-ratiometric fluorescence in SA	UV photobleaching susceptibility	[300]

**Table 11:** Summary of quantitative performance metrics for recent representative alginate-based hydrogels used in food preservation and packaging systems.

Hydrogel System	Primary Function	Active Component	Preservation Mechanism	Shelf Life Extension	Biodegradability	Ref.
Corn Starch/SA Emulsion	Emulsion stabilization	30% oil content	Structural stabilization	Extended	High	[292]
Anthocyanin-loaded SA Beads	Freshness indicator	C3G: 0.047–0.056 mg/g	pH-sensitive color change	>4 weeks (4 °C)	Biodegradable	[301]
Chitosan/CQDs/SA	Multifunctional packaging	Carbon quantum dots	pH-responsive fluorescence	Extended	Biodegradable	[302]
Glycerol/NaCl/SA	Rapid detection	Active components optimized	Chemical sensing	<2 min	Biodegradable	[64]

**Table 12:** Application suitability matrix: preparation type selection based on application requirements and performance criteria.

Application	Ionic	Covalent	Hybrid	Physical	Recommended
Drug Delivery	Excellent	Good	Excellent	Fair	Hybrid/Ionic
Cell Encapsulation	Excellent	Fair (toxicity)	Excellent	Good	Hybrid/Ionic
Tissue Engineering (load-bearing)	Fair	Excellent	Excellent	Poor	Covalent/Hybrid
Wound Healing	Excellent	Good	Excellent	Fair	Hybrid/Ionic
3D Bioprinting	Good	Fair	Excellent	Fair	Hybrid
Environmental Remediation	Excellent	Good	Good	Excellent	Ionic/Hybrid
Flexible Devices	Fair	Fair	Excellent	Good	Hybrid
Food Safety	Excellent	Fair	Excellent	Good	Ionic/Hybrid

## Data Availability

Not applicable.
